# Authigenic mineralization in Surtsey basaltic tuff deposits at 50 years after eruption

**DOI:** 10.1038/s41598-023-47439-4

**Published:** 2023-12-21

**Authors:** Giovanna Montesano, Concetta Rispoli, Paola Petrosino, Marie D. Jackson, Tobias B. Weisenberger, Magnús T. Gudmundsson, Piergiulio Cappelletti

**Affiliations:** 1 Dipartimento di Scienze della Terra, dell’Ambiente e delle Risorse, Università degli Studi di Napoli Federico II, Complesso Universitario Monte Sant’Angelo, Ed. 10, Via Cintia 26, 80126 Naples, Italy; 2 Department of Geology and Geophysics, University of Utah, Salt Lake City, UT USA; 3 Institute of Earth Sciences, University of Iceland, Reykjavík, Iceland; 4 Helmholtz Centre Potsdam - GFZ German Research Centre for Geosciences, Potsdam, Germany

**Keywords:** Geology, Mineralogy

## Abstract

Alteration of basaltic glass and in situ mineral growth are fundamental processes that influence the chemical and material properties of Earth’s oceanic crust. These processes have evolved at the basaltic island of Surtsey (SW Iceland) since eruptions terminated in 1967. Here, subaerial and submarine lapilli tuff samples from a 192 m-deep borehole drilled in 2017 (SE-02b) are characterized through petrographic studies, X-ray powder diffraction analyses, and SEM–EDS imaging and chemical analyses. The integrated results reveal (i) multi-stage palagonitization processes in basaltic glass and precipitation of secondary minerals from matrix pore fluids, (ii) multi-stage crystallization of secondary phillipsite, analcime and Al-tobermorite in the vesicles of basaltic pyroclasts and (iii) variations in palagonitization processes as a function of thermal and hydrological domains. Although temperature appears to be an important factor in controlling rates of secondary mineralization, the chemistry of original basaltic components and interstitial fluids also influences reaction pathways in the young pyroclastic deposits. The integration of systematic mineralogical analyses of the 50-year-old tuff from one of the most carefully monitored volcanic sites on Earth, together with temperature monitoring in boreholes since 1980, provide a reference framework for evaluating mineralogical evolution in other Surtseyan-type volcanoes worldwide.

## Introduction

The emplacement of pyroclastic and volcaniclastic deposits during episodes of active volcanism is commonly followed by syn- and post-depositional minerogenetic processes, resulting in newly formed mineral phases, such as zeolites and clay minerals. Secondary minerals, in particular zeolites, are formed by the transformation of parent volcanic glass during interaction with alkaline solutions^1^ in diverse marine and continental environments^2–4^. Since emerging from the seafloor in 1963, the island of Surtsey has provided a pristine natural research laboratory for monitoring submarine pyroclastic eruptions and the formation and development of an oceanic island, tracing the geological and biological history of surficial basaltic deposits^5–9^, and deciphering the hydrothermal alteration of basaltic tephra, including zeolite mineralization, in a drill core acquired in 1979 from subaerial and submarine deposits^10–13^.

The Surtsey Underwater Volcanic System for Thermophiles, Alteration processes, and Innovative concretes (SUSTAIN) drilling project at Surtur crater, sponsored by the International Continental Scientific Drilling Program (ICDP), retrieved three drill cores that transected the 25–141 °C hydrothermal system in 2017^14–16^. Studies of these cores and of the 1979 core^10,11^ provide new insights into the hydrothermal, geochemical, geomagnetic, and microbiological processes that have occurred in the basaltic tephra and intrusive basalt since explosive and effusive eruptions ceased in 1967.

Studies of drill core samples describe rates of time-lapse hydrothermal alteration in lapilli tuff^10,11,17^, authigenic mineral texture^13^, and new insights into the structure of Surtsey^18,19^. Geochemical studies of the geothermal system describe an analogue for water chemistry and chemical fluxes in low temperature Mid-Ocean Ridge (MOR) environments^20^ as well as element mobilities associated with palagonitization of basaltic glass^21^.

**Figure 1:**
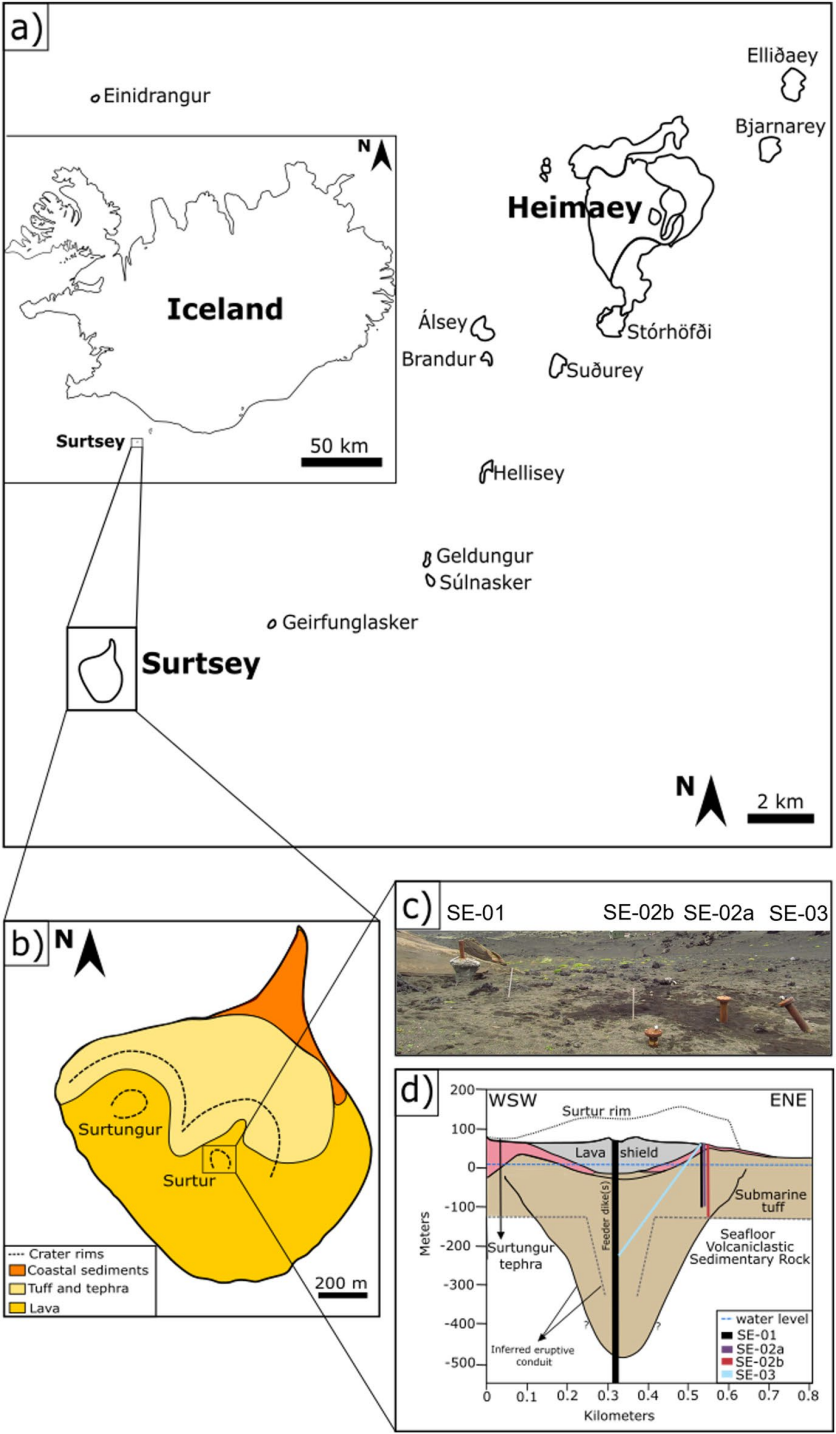
Surtsey volcano, Iceland. (**a**) Sketch map of the Vestmannaeyjar archipelago with Surtsey at its southwestern tip; (**b**) simplified sketch map of Surtsey showing the main geo-lithological units and crater rims (dashed lines) of Surtur and Surtungur (modified after^22^); (**c**) field photograph showing wellheads of the three 2017 and 1979 boreholes; (**d**) interpretative cross-section of Surtur showing the original crater rim (at the age of its formation; dotted line), 1979 (SE-01) and 2017 (SE-02a, SE-02 b and SE-03) cored drill holes (after^14,18^), pyroclastic deposits, lava shield in the central crater, and seafloor volcaniclastic sedimentary rock. The subseafloor inferred eruptive conduit: black solid line^23^ and minimum expression (gray dashed line)^14^. *Source* (**a,b,d**) generated with Inkscape software 1.0.2 (https://inkscape.org/release/inkscape-1.0.2/).

**Figure 2:**
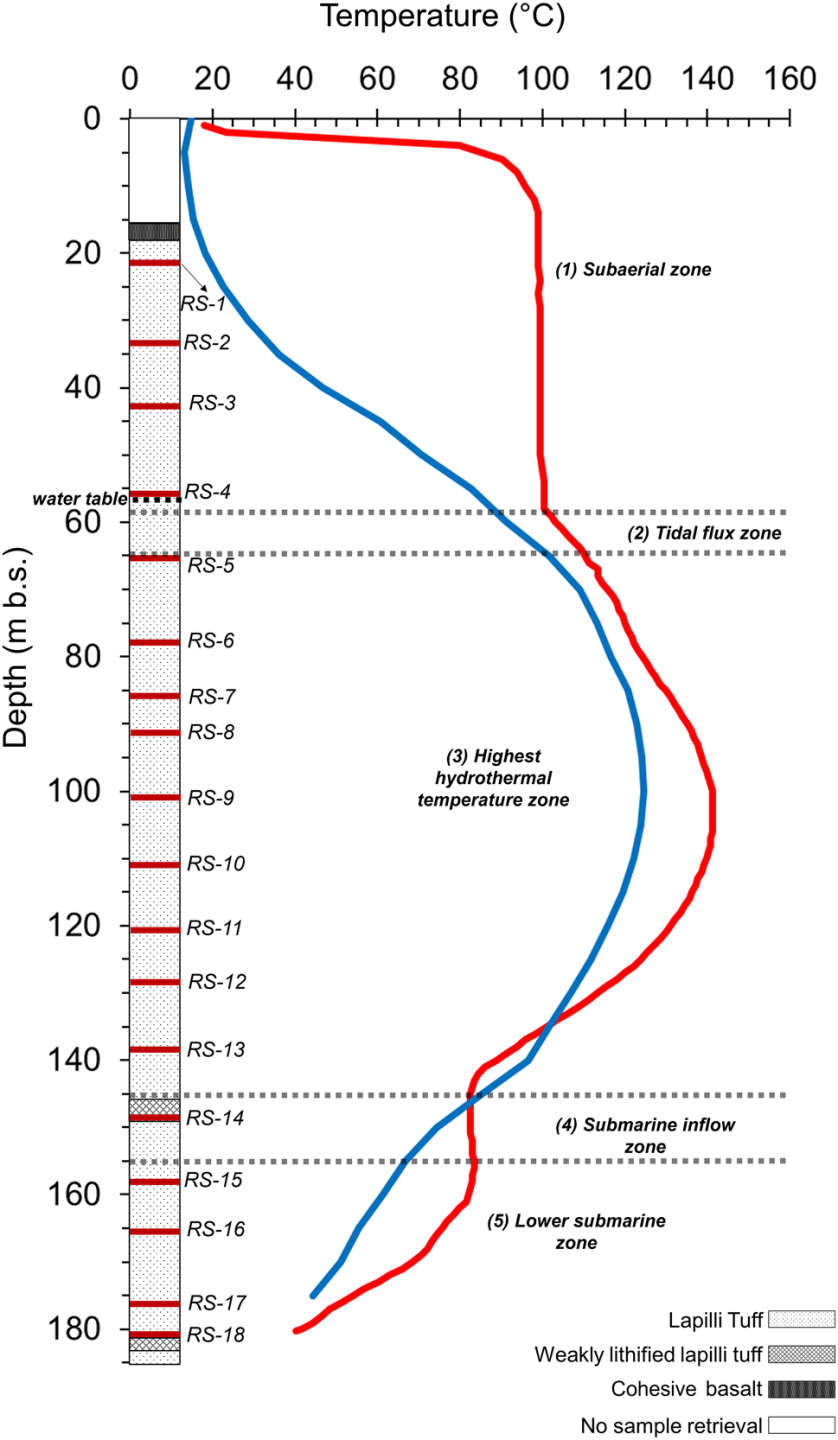
Distribution of reference samples, RS-1 to RS-18, along the SE-02b drill core and the 1980 and 2017 temperature profiles measured in borehole SE-01. Structural and hydrothermal zones of Surtur crater as reported in Table 1. The temperature log data are derived from 1980 measurement (carried out by the National Energy Authority; light red line) after^10,11,33^; 2017 temperature log data (dark blue line) after^14,15^.

By contrast, this article describes the mineralogy and crystal-chemistry of authigenic and secondary phases, their stratigraphic distribution within the subaerial and submarine volcanic structure of Surtur (Fig. 1b,d), and their paragenetic relationships with weakly-altered basaltic glass. Secondary minerals are here considered to precipitate from interstitial fluids in pores or vesicles while authigenic minerals form within pre-existing glass, amorphous or crystalline phases^17^. A suite of reference samples, designated in 2017 as collaborative materials for interdisciplinary research within the SUSTAIN science team^14^, are described with various fine-scale analyses (Table 1, Fig. 2). The purpose of this research is to further reconstruct the diverse and interdependent mechanisms that led to the consolidation of freshly erupted tephra and the lithification of the lapilli tuff that currently make up the island of Surtsey. The integrated analytical results provide a key reference for assessing the alteration processes and thermal history of oceanic volcanic deposits, including textural and chemical guideposts in secondary and authigenic mineral assemblages that elucidate fluid evolution over time^3^.

**Table 1:** Investigated samples with corresponding depths and macroscopic descriptions.

Drill hole	Depth (m b.s.)	Hydrothermal zone	Sample	IGSN #	Colour	Features
SE-02b	22.6	1	RS-1	ICDP5059EXZ3701	Mottled brown-dark gray	Medium armored lapilli tuff
SE-02b	34.6	1	RS-2	ICDP5059EX04701	Brown	Medium to fine lapilli tuff with rare, armored lapilli
SE-02b	43.7	1	RS-3	ICDP5059EX34701	Blackish green	Medium lapilli tuff with armored lapilli
SE-02b	56.0	1	RS-4	ICDP5059EX44701	Mottled brown-dark gray	Medium lapilli tuff with armored lapilli
SE-02b	65.3	2	RS-5	ICDP5059EX54701	Dark green	Medium lapilli tuff
SE-02b	78.2	3	RS-6	ICDP5059EX64701	Dark gray	Medium lapilli tuff
SE-02b	86.4	3	RS-7	ICDP5059EX74701	Green	Medium lapilli tuff
SE-02b	92.6	3	RS-8	ICDP5059EX84701	Green	Medium to fine lapilli tuff
SE-02b	101.5	3	RS-9	ICDP5059EX94701	Green	Medium lapilli tuff
SE-02b	110.9	3	RS-10	ICDP5059EXA4701	Green	Medium lapilli tuff
SE-02b	120.6	3	RS-11	ICDP5059EXB4701	Green	Medium lapilli tuff
SE-02b	128.0	3	RS-12	ICDP5059EXC4701	Green	Medium lapilli tuff
SE-02b	138.4	3	RS-13	ICDP5059EXD4701	Green	Medium lapilli tuff
SE-02b	148.7	4	RS-14	ICDP5059EXE4701	Black	Medium lapilli tuff
SE-02b	157.4	5	RS-15	ICDP5059EXF4701	Green	Medium lapilli tuff
SE-02b	165.6	5	RS-16	ICDP5059EXG4701	Green	Medium lapilli tuff
SE-02b	176.1	5	RS-17	ICDP5059EXH4701	Dark green	Medium lapilli tuff
SE-02b	180.9	5	RS-18	ICDP5059EXI4701	Blackish brown	Medium lapilli tuff

Numbers (1 to 5) indicate structural and hydrothermal zones of Surtur crater [(1) subaerial zone, (2) tidal flux zone, (3) highest hydrothermal temperature zone, (4) submarine inflow zone and (5) lower submarine zone] as reported by Refs.^10,11,16–21,32^.

Clast sizes: medium lapilli: 8 to 10 mm (see^16^ for further details).

*IGSN* International Generic Sample Number.

## Surtsey geological setting and hydrothermal system

Surtsey is located in the southernmost sector of the Vestmannaeyjar archipelago, which forms the offshore extension of the Iceland SE rift zone (Fig. 1a). The island grew from a seafloor depth of ~ 130 m during explosive and effusive basaltic eruptions in 1963–1967^10,11,24,25^. The primary geological units, tephra, lava and intrusive basalt, have an alkali basalt composition with phenocrysts of olivine, plagioclase and Cr-spinel^12^. Visible steam rising from the tephra pile, observed in 1967, indicated an active hydrothermal system. One year later, the first signs of palagonitization of basaltic glass in surficial tephra were recorded^26^. There, the original glass fraction was chemically modified and hydrated so that release of chemical elements to the surrounding fluids initiated precipitation of secondary minerals. These minerals partially filled voids in the tephra deposits and also contributed to tephra consolidation and lithification^11,13,21,26,27^. The palagonitized lapilli tuff exhibited higher mechanical resistance to marine erosion compared to the unconsolidated tephra. Thus, lithification of the tephra deposits produced by diverse hydrothermal alteration processes increased the erosional resistance of the newly formed island, enhancing its relative stability and longevity^26–28^.

Investigations of samples from the 181 m, vertical 1979 core (SE-01), situated on the eastern edge of the Surtur tuff cone (Fig. 1b,d), described the composition of the loose tephra and lapilli tuff, rates of alteration and consolidation, as well as an authigenic smectitic clay, secondary zeolite, and Al–tobermorite mineral assemblage^10,17^. Al–tobermorite is an unusual calcium–silicate hydrate mineral that is the principal mineral cement in ancient Roman marine concretes^29,30^. X-ray microdiffraction studies describe the transition of fresh basaltic glass to nano-crystalline clay mineral^13^ during post-depositional alteration processes. Petrographic studies describe altered glass with variable optical properties and textures, ranging from a translucent, reddish-brown gel-like phase in plane-polarized light (PPL) (type I) to cryptocrystalline, strongly birefringent fibro-palagonite, yellow in PPL (type II), and dark, grainy palagonite, almost opaque in PPL (type III)^17^.

In 2017, the SUSTAIN project acquired two vertical cores (SE-02a and SE-02b) and one core inclined 33.4° from vertical, (SE-03) (Fig. 1c,d), drilled through the still hot volcano (maximum temperatures of 124 degrees C, SE-02b; 141 degrees C, SE-03)^14,15^. Boreholes SE-02a and SE-02b were drilled to 152 and 192 m depth below the surface (m b.s.), respectively, while borehole SE-03 reached a vertical depth of approximately 295 m b.s. and 354 m measured depth^33^. None of the boreholes transected the pre-eruption seafloor, presumably located at a depth of 190–195 m b.s.^14,15,31^.

## Materials and methods

**Figure 3:**
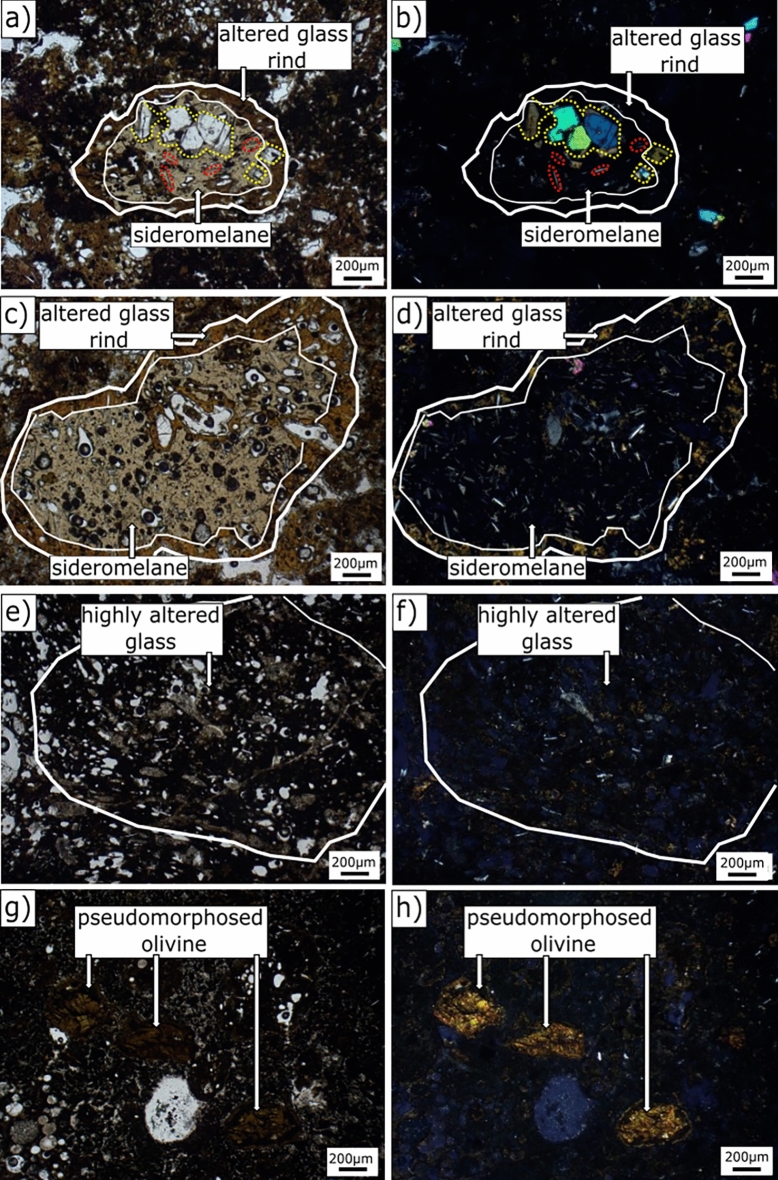
Petrographic images of the three different types of glass fabrics (apparent sideromelane, altered glass rinds and highly altered glass) and complete pseudomorphic olivine replacement by clay-like phases [*PPL* plane-polarized light (left) and *CPL* cross-polarized light (right)]. (**a,b**) RS-1 (22.6 m b.s.); (**c,d**) RS-2 (34.8 m b.s.); (**e,f**) RS-9 (101.5 m b.s.); (**g,h**) RS-10 (110.9 m b.s.). See Figs. 1, 2 and Table 1 for descriptions of hydrothermal and structural zones. Mineral abbreviations from Ref.^38^. *php* phillipsite, *anl* analcime, *tbm* Al-tobermorite, *gp* gypsum, *cal* calcite.

The samples analysed in the present research are part of a reference set of samples from the 2017 SE-02b drill core. To date, open access datasets have been published for whole rock major element geochemistry, magnetic properties, rock water absorption, bulk density^14^ and X-ray µ-computed tomography reconstructions^34^. Petrographic, mineralogical, and chemical analyses were performed on reference samples (RS) of lapilli tuff from SE-02b vertical borehole (see Fig. S1 in Supplementary Information) at the Department of Earth, Environmental and Resources Sciences (DiSTAR) of the University of Naples Federico II. An overview of the samples, with corresponding depths and descriptions of general macroscopic features, as well as distribution along the SE-02b drill core, is given in Table 1, Figs. 2 and 3.

Petrographic analyses were carried out on thin sections through polarized optical microscopy (POM) with a LEITZ Laborlux 12 Leica POL polarizing microscope.

Qualitative and quantitative phase analyses were performed by means of X-Ray Powder Diffraction (XRPD and QXRPD, respectively) using a Malvern Panalytical X’Pert Pro diffractometer equipped with a RTMS X’Celerator. Malvern Panalytical HighScore Plus 3.0 software was used for phase identification, along with PDF-2 and ICSD database (International Crystal Structure Database-ICSD, 2012)^35^. Operating conditions were: CuKα radiation, 40 kV, 40 mA, 2θ range from 4° to 70°, equivalent step size 0.017° 2θ, equivalent counting time 120 s per step. Data sets were analyzed using RIR/Rietveld method^36,37^ and TOPAS 5 software (BRUKER AXS Company). Powders with grain size < 10 µm were obtained using a McCrone micronizing mill (agate cylinders and wet grinding time of 15 min). An α-Al_2_O_3_ internal standard (1 µm, Buehler Micropolish) was added to each sample at a rate of 20 wt%. Starting atomic coordinates for Rietveld analyses were taken from literature (ICSD; International Crystal Structure Database-ICSD, 2012^35^), a background profile was fitted using a Chebyshev polynomial function with variable number of coefficients (5–12); diffraction peak profiles were modeled refining crystallite size and strain (Lorentzian contribution) coefficients and two Gaussian coefficients. Unit cell parameters along with weight fractions were also refined. PO (preferred orientation) was treated with a March-Dollase approach^39^, as needed.

Microtextural observations and quantitative microanalyses were carried out by Field Emission Scanning Electron Microscopy Zeiss Merlin coupled with an Energy Dispersive Spectroscopy (FESEM/EDS). Measurements were performed with an INCA X-stream pulse processor (using a 15 kV primary beam voltage, 50–100 µA filament current, from 30,000 to × 200,000 magnification, 20 mm WD and 10 s net acquisition real time). The INCA Energy software was employed using the XPP matrix correction scheme and the pulse pile up correction. The quant optimization was carried out using cobalt (FWHM—full width at half maximum peak height of the strobed zero = 60–65 eV). Smithsonian standards used for calibration are reported in Ref.^40^. The precision and accuracy of EDS analyses are reported in Ref.^41^.

Thermodynamic calculations were conducted using The Geochemist’s Workbench software package (GWB, Standard release 6.0^42^). The ACT2 component of GWB was used for activity-activity diagrams, implementing internal database^43^. H_2_O and silica activity values were chosen to be consistent with the investigated system (log *a*[H_2_O] = 1, log *a*[SiO_2_(aq)] = 10^−4.1^)^1^.

## Results

Analytical data are described in the following sections, with a particular focus on the compositional variations of the mineral phases as a function of depth and the different hydrothermal zones (see Table 1, Fig. 2).

### Petrographic microscopy

The samples are moderately to strongly vesicular tuffs consisting of inequigranular hypo-crystalline lapilli, angular to slightly rounded in shape, engulfed in a fine and coarse-ash matrix locally containing loose plagioclase and olivine microcrystals. The larger pyroclasts, as well as the ash matrix, appear extensively altered. Juvenile fragments show vesicles of variable size and shape, commonly filled by authigenic minerals.

#### Sideromelane, primary volcanic crystals and their alteration

A translucent, sideromelane-like phase occurs mainly in weakly-consolidated tephra and lapilli tuff deposits in the subaerial zone and the lower submarine zone, near the pre-eruption seafloor (zones 1 and 5; e.g., RS-1, Fig. 3a,b) and, occasionally, in the high-temperature hydrothermal zone. Although sideromelane is defined as fresh glass^44^, µXRD studies show that very little fresh glass persists in archived samples of the 1979 drill core; furthermore, domains of glass alteration occur at a sub-micrometer scale^13^. Here, sideromelane refers to a gel-like phase in vesicular pyroclasts that is pale yellowish-grey in PPL and entirely isotropic in cross-polarized light (CPL); it is henceforth reported in quotes as “apparent sideromelane”. The identification of sideromelane is thus based solely on optical petrographic criteria and does not consider the potential occurrence of authigenic phases at the sub-micrometer scale. Euhedral lath-shaped plagioclase and anhedral to subhedral olivine crystals (Fig. 3a–d), are ubiquitous primary phases. They occur both as phenocrysts and microphenocrysts within the altered fragments of volcanic glass and as loose crystals in the altered fine-ash matrix.

Three principal palagonitic textures (Fig. 3a–f), as previously described by Ref.^17^, are present. Optically isotropic palagonitic rinds (e.g., Fig. 3a,b) are sporadically present mainly in the uppermost (RS-1) and the lowermost (RS-18) samples of borehole SE-02b (zone 1 and 5, respectively).

The fibrous palagonitic type is present in all samples (e.g., RS-2, Fig. 3c,d), except for samples RS-1, RS-8 and RS-18. The granular, opaque palagonitic-type occurs through the length of the drill core (e.g., RS-9, Fig. 3e,f) except in samples RS-4 and RS-5, near the zone of tidal flux. Types 2 and 3 may transition into each other, occuring as alteration rinds of variable thickness around once glassy pyroclasts or fully obliterating the original basaltic glass.

Olivine crystals persist within zones 4 and 5, the lowermost deposits below the submarine inflow zone. They show clay-like alteration rims of variable thickness, either regular, following the original crystal boundaries, or irregular and denticulate, directed towards the interiors of the crystals. Elsewhere, almost complete pseudomorphic olivine replacement by clay-like secondary minerals (e.g., in RS-10, Fig. 3g,h) is common (zones 1, 3 and 5).

#### Newly formed phases

**Figure 4:**
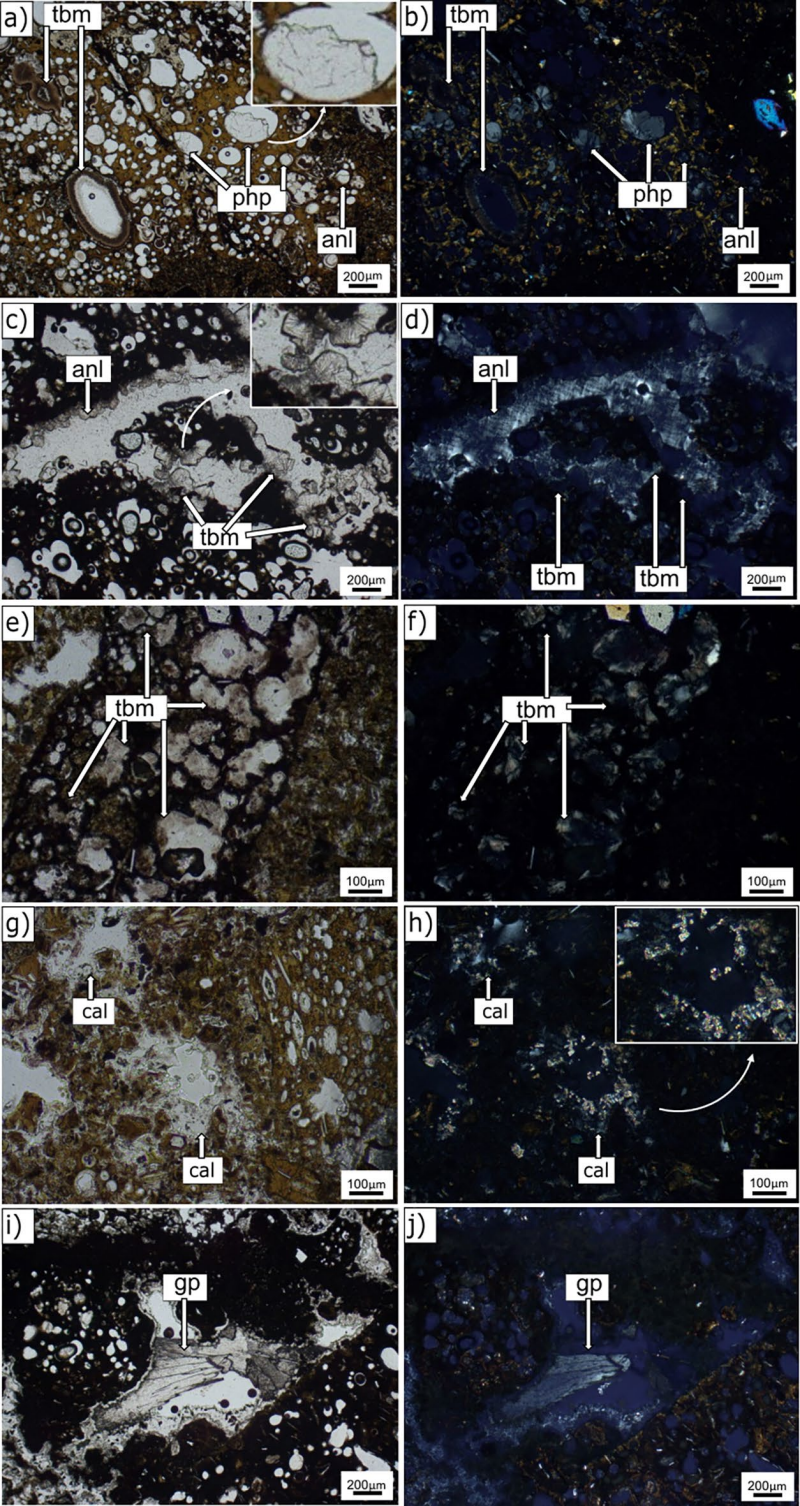
Petrographic images of authigenic mineral surface coatings [*PPL* plane-polarized light (left) and *CPL* cross-polarized light (right)]. **(a,b)** RS-14 (148.7 m b.s.); **(c,d)** RS-2 (22.6 m b.s.); **(e,f)** RS-3 (43.7 m b.s.); **(g,h)** RS-3 (43.7 m b.s.); **(i,j)** RS-9 (101.5 m b.s.). See Figs. 1, 2 and Table 1 for descriptions of hydrothermal and structural zones. Mineral abbreviations from Ref.^38^: *php* phillipsite, *anl* analcime, *tbm* Al-tobermorite, *gp* gypsum, *cal* calcite.

Secondary phases occur as zeolite, calcium–aluminium–silicate hydrate, carbonate, and sulphate minerals. They are present as fillings in pyroclast vesicles and pores of the binding fine-ash matrix, as well as surface coatings in large pores of the altered ash matrix (Fig. 4a–j). Clay-like minerals are present as well, as glass and olivine alteration. Figure 4 shows petrographic images of typical alteration microenvironments in basaltic pyroclasts and matrix pores. Euhedral analcime typically forms coatings on the inner walls of vesicles of coarse ash or lapilli (Fig. 4a,b, RS-14) or along void spaces in the binding fine-ash matrix (Fig. 4c,d, RS-2). Phillipsite in vesicles (Fig. 4a,b, RS-14) appears as colourless intergrowths in PPL and radiating prismatic crystals (0.1 to 0.25 mm), with moderate birefringence and low interference colours in CPL.

Al-tobermorite in vesicles appears as acicular microcrystals arranged in discrete fan-shapes (Fig. 4c,d, RS-2). Tightly bound needles of Al-tobermorite also formed (Fig. 4a,b,e,f, RS-14 and RS-3, respectively) in the subaerial tuff and submarine inflow zones.

Highly birefringent, microcrystalline calcite forms scattered coatings along the inner walls of matrix pores (Fig. 4g,h, RS-3). Colourless, prismatic to fibrous radiating gypsum crystals show low birefringence and light grey interference colours (Fig. 4i,j, RS-9). The crystals commonly fill larger, irregularly-shaped voids within the binding matrix mainly in RS-3, RS-4, RS-9, and RS-12.

### Scanning electron microscopy

#### Sideromelane and altered glass

**Figure 5:**
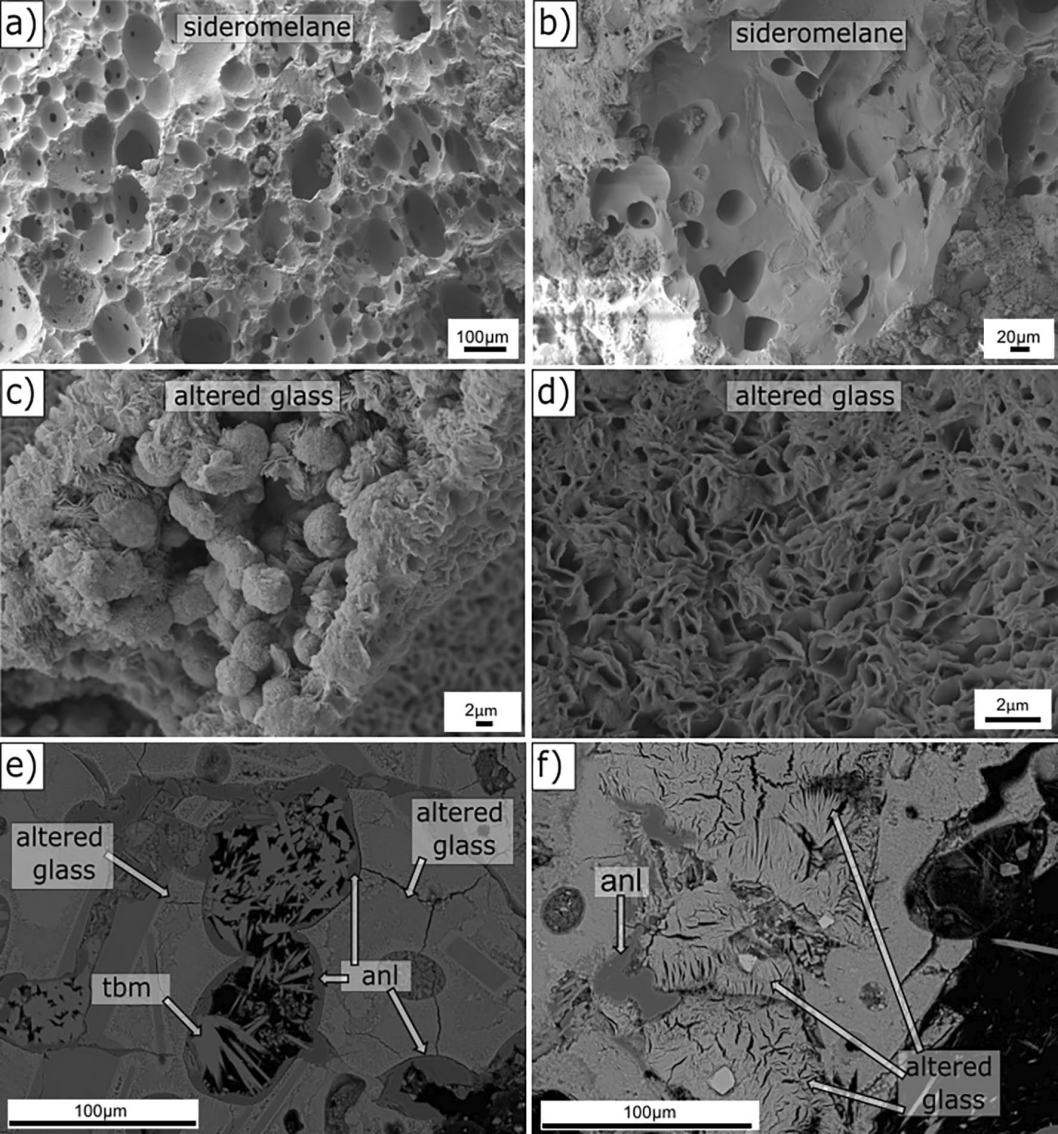
SEM images of alteration features in pyroclasts. **(a,b)** SEM-SE images of sideromelane, RS-18 (180.9 m b.s.); **(c)** SEM-SE images of curled-edge features in altered glass, RS-8 (92.6 m b.s.); **(d)** SEM-SE image of flake-like features in highly altered glass, RS-8 (92.6 m b.s.); **(e)** SEM-BSE image of altered glass, analcime and tobermorite, RS-2 (34.8 m b.s.); **(f)** SEM-BSE image of altered glass and analcime, RS-8 (92.6 m b.s.). See Figs. 1, 2 and Table 1 for hydrothermal and structural zones. Mineral abbreviations from Ref.^38^, *anl* analcime, *tbm* Al-tobermorite.

SEM-SE images of “sideromelane” show a typical homogeneous, texturally uniform and smooth surface^45^ with abundant rounded, spherical to elliptical vesicles, commonly coalescent (Fig. 5a,b, RS-18). By contrast, domains of altered glass display a wide range of features reflecting progressive degrees of alteration within the original vesicular morphology, shown in thin section (Fig. 3a–f). In SEM-SE images, altered basaltic glass appears in globular micro-morphological arrangements (Fig. 5c, RS-8) or as tiny plates with curled edges or feathered “flakes” of randomly intergrown smectite-like clay minerals (Fig. 5d, RS-8). The globules, often agglutinate, are about 3 μm in diameter. Morphological features of the altered glass are highly heterogeneous and may locally display a ragged or sponge-like surface (Fig. 5e, RS-2), or a distinct fibrous appearance, typically with fan or leaf-shaped textures in altered olivine crystals (Fig. 5f, RS-8).

**Figure 6:**
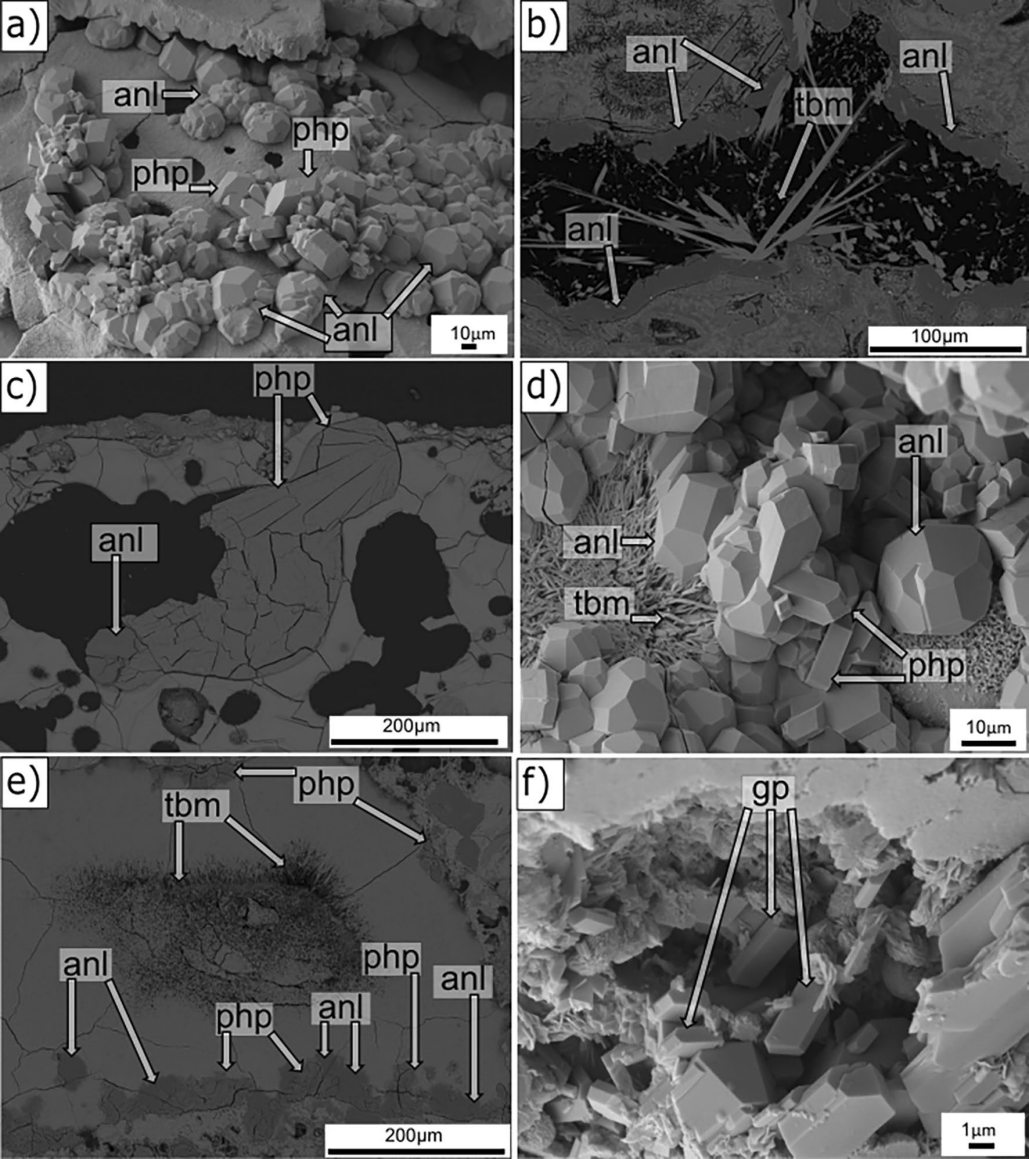
SEM images showing secondary mineral growth in pyroclasts **(a–e)** and a matrix pore **(f)**, **(a,d,f)** SEM-SE and **(b,c,e)** SEM-BSE images. **(a,c,d)** RS-3 (43.7 m b.s.); **(b)** RS-17 (176.0 m b.s.); **(e)** RS-14 (148.7 m b.s.); **(f)** RS-9 (101.5 m b.s.). See Figs. 1, 2 and Table 1 for descriptions of hydrothermal and structural zones. Mineral abbreviations from Ref.^38^, *php* phillipsite, *anl* analcime, *tbm* Al-tobermorite, *gp* gypsum.

Figure 6a-e shows typical alteration environments in the basaltic pyroclasts. Analcime appears as discrete, well-defined euhedral crystals nestled in the inner walls of vesicles (Fig. 6a, RS-3), here with euhedral phillipsite crystals. Analcime is also commonly associated with Al-tobermorite **(**Figs. 5e, RS-2, 6b, RS-17). Phillipsite occurs as well-defined crystals filling vesicles and displaying a rosette morphology, or replaced by analcime (Fig. 6c, RS-3).

Groups of randomly oriented, sprays of Al-tobermorite appear to postdate analcime and phillipsite (Fig. 6b, RS-17, d, RS-3, e, RS-14). Gypsum crystals (Fig. 6f, RS-9) have platy to coarse prismatic forms in matrix pores, with larger crystals commonly below water level.

### X-ray powder diffraction

**Figure 7:**
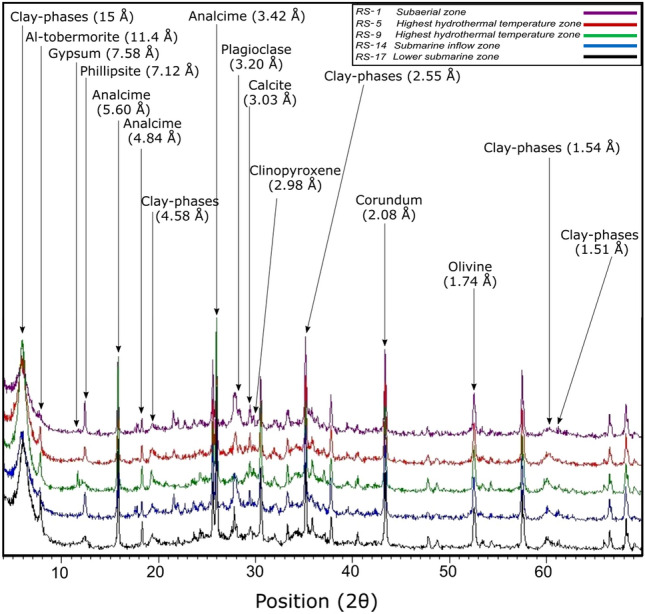
Representative XRPD patterns of the subaerial RS-1 and submarine RS-5, RS-9, RS-14 and RS-17 lapilli tuff samples from the 2017 SE-02b drill core.

QXRPD results are reported in Table 2 and representative XRPD patterns are shown in Fig. 7.

**Table 2:** QXRPD analyses of selected lapilli tuff samples from the 2017 SE-02b drill core.

Quantitative XRPD											
Sample	Depth (m)	Zone	Pl	Ol	Cpx	Anl	Cal	Php	Gp	Tbm	AC
RS-1	22.6	1	11	3	3	12	2	10	0	2	57
RS-3	43.7	1	11	3	4	17	2	2	1	4	56
RS-5	65.3	2	6	2	5	17	2	6	0	4	58
RS-6	78.2	3	6	1	4	17	2	6	1	5	58
RS-8	92.6	3	4	1	7	21	2	4	1	5	55
RS-9	101.5	3	5	1	7	24	2	1	1	5	54
RS-10	110.9	3	6	1	7	22	2	1	1	5	55
RS-12	128.0	3	4	1	8	26	2	1	1	4	53
RS-14	148.7	4	12	2	4	12	2	8	0	3	57
RS-17	176.1	5	6	2	5	17	2	6	0	4	58

Mineral abbreviations, where applicable, from Ref.^38^. Hydrothermal and structural zones as reported in Fig. 2 and Table 1.

*AC* amorphous content and disordered clay minerals, *pl* plagioclase, *cpx* clinopyroxene, *ol* olivine, *php* phillipsite, *anl* analcime, *tbm* Al-tobermorite, *cal* calcite, *gp* gypsum.

In the lapilli tuff samples selected for QXRPD analysis (Table 2) (i) plagioclase content ranges between 4 and 12 wt%, (ii) olivine is present up to 3 wt%, and (iii) clinopyroxene varies from 3 to 8 wt%. Note, however, that much of the original olivine has undergone authigenic alteration. The QXRPD analysis also calculates an “amorphous” content of 53–60 wt% with an average of 57 wt% for these samples. This comprises the small amount of fresh glass that may persists in some deposits as well as the mass percentage of nanocrystalline phases with low long-range order^13^ and/or poorly-ordered phases such as smectite-like clay mineral(s).

The reflections at 1.54 Å and 1.51 Å correspond to the main d_060_ reflection of tri- and di-octahedral clay mineral species^46^. The principal secondary crystalline phases include phillipsite (1 to 10 wt%), analcime (12 to 24 wt%) and Al-tobermorite (2 to 5 wt%). Approximately 2 wt% of calcite, and gypsum (1 wt%) are detected.

### Chemical compositions of sideromelane and altered glass

**Figure 8:**
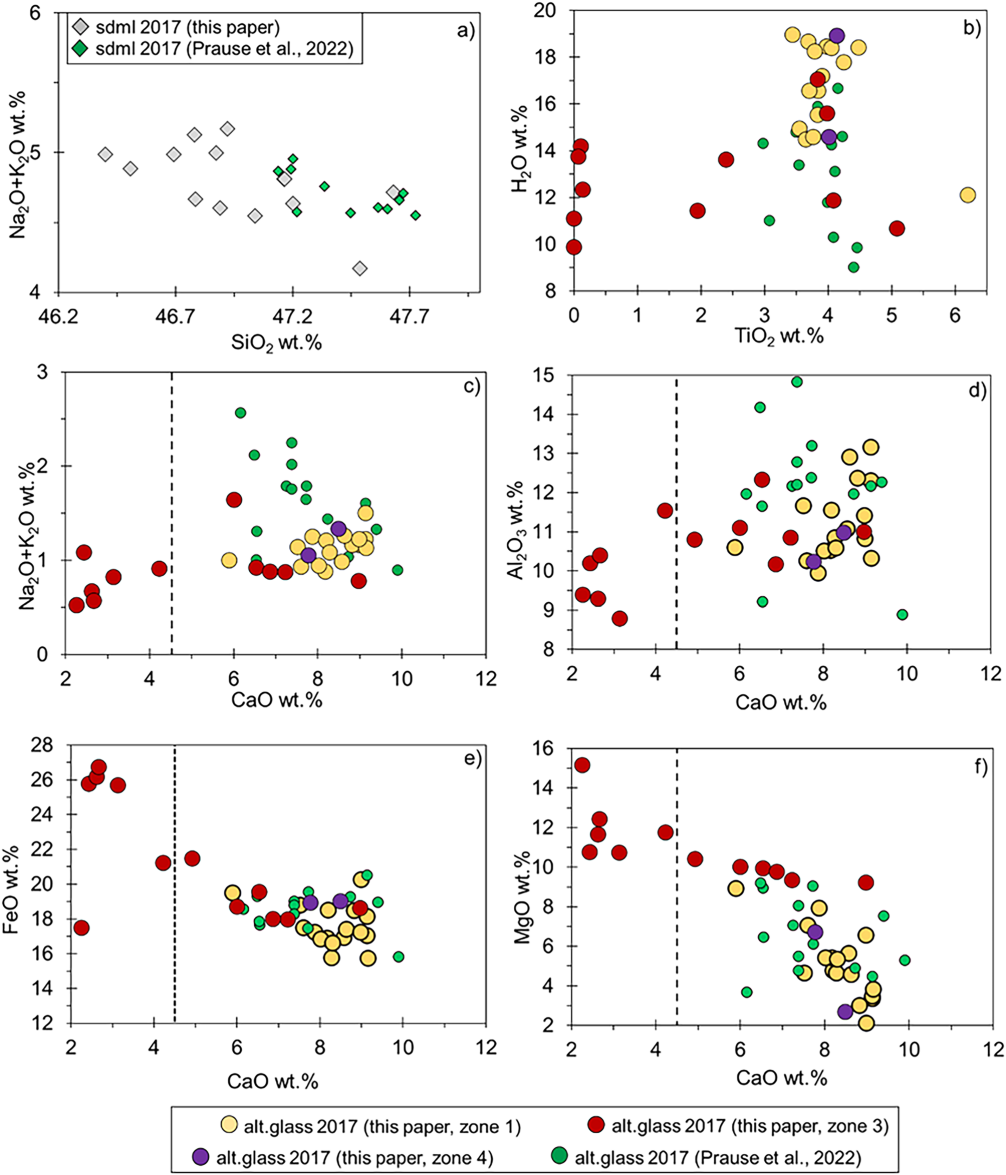
SEM–EDS analyses for apparent sideromelane (diamonds) and variably altered glass (yellow, red and violet circles for zones 1, 3 and 4 respectively) for representative Surtur lapilli tuff from 2017 drill core samples. Apparent sideromelane = **(a)** Na_2_O + K_2_O vs. SiO_2_ (normalized to 100); Altered glass = **(b)** H_2_O vs. TiO_2_; **(c)** Na_2_O + K_2_O, **(d)** Al_2_O_3_, **(e)** FeO, and **(f)** MgO vs. CaO. Data for other 2017 core analyses^21^ are shown for comparison. Dashed vertical lines separate the analyses of high-CaO and low-CaO altered glass. Colours refer to hydrothermal and structural zones (Fig. 2, Table 1).

The results of SEM–EDS analyses of primary and authigenic phases in microenvironments similar to those in Figs. 4, 5 and 6 reveal substantial compositional and mineralogical variations within the SE-02b lapilli tuff samples (Fig. S1). Primary volcanic crystals are mainly olivine (Fo_74–81_; Fig. S2) and plagioclase (An_64–70_; Fig. S3). Only a few samples, RS-2 (22.6 m b.s.), RS-3 (43.7 m b.s.), RS-14 (148.7 m b.s.) and RS-18 (190.4 m b.s.) contain apparent sideromelane (Table S1) with SiO_2_ from 46.4 to 47.6 wt% and alkali from 4.2 to 5.2 wt% (Fig. 8a). Note that, given the unavailability of bulk unaltered basaltic tephra, the EDS compositional data of microlite-free apparent sideromelane are used to infer the chemical composition of the juvenile fraction of the lapilli tuff.

The individual point compositions of flaky or globular features in altered basaltic glass [Fig. 8b–f (H_2_O vs. TiO_2_, Na_2_O + K_2_O, Al_2_O_3_, FeO and MgO vs. CaO)] define a high-CaO group (CaO > 4.5 wt%) and a low-CaO group (CaO < 4.5 wt%) (Table S2), apparently in response to progressively greater alteration. The binary plot of Fig. 8b shows the H_2_O content of altered glass (obtained by difference) vs. TiO_2_, which is a relatively immobile element. The TiO_2_ content of the high-CaO group is similar to that of apparent sideromelane, while the TiO_2_ content of the low-CaO group is lower, overall. Low-Ca group analyses resemble the compositions of saponite-like smectite trioctahedral clay minerals, in which TiO_2_ is almost completely absent^47,48^.

Alkali content, Na_2_O + K_2_O wt%, is relatively constant (0.9 to 1.5 wt%, yellow and purple circles) in samples of zones 1 and 4, the subaerial tuff and the weakly-consolidated tuff of the submarine inflow zone, but is more variable (0.5 to 1.6 wt%, red circles) in samples of zone 3, the higher temperature hydrothermal zone (Fig. 8c). Al_2_O_3_, FeO and MgO wt% show even more scattering, when referenced to CaO content (Fig. 8d–f).

With respect to apparent sideromelane (Fig. 8a, Table S1), analyses of altered glass from all the structural zones exhibit lower Al_2_O_3_ and alkali overall, but also lower SiO_2_ and CaO (Tables S1, S2). Therefore, differences between altered glass vs. apparent sideromelane are (i) 3.1–9.1 wt% vs. ~ 16 wt% for Al_2_O_3_, (ii) 1 to 2 wt% vs. 4–5 wt% for Na_2_O + K_2_O, (iii) 32.9–41.0 wt% vs. ~ 46 wt% for SiO_2_, (iv) 2.2–9.1 wt% vs. ~ 10–12 wt% for CaO. On the contrary, FeO wt% in altered glass is higher than in apparent sideromelane (18.9 wt% vs. ~ 13.3 wt%). MgO is overall higher in altered glass (up to 15.1 wt% vs. 5.4–6.2 wt% for apparent sideromelane), with a few exceptions for altered glass (2.1–4.6 wt%) in samples from the subaerial and submarine inflow zones.

### Crystal chemistry of secondary minerals

Alteration of basaltic glass and crystallization of secondary minerals has been described in the subaerial and submarine deposits at Surtsey, within the 1979 SE-01 core^11,13^ and in the 2017 SE-02a and SE-02b cores^17,21^.

Little information exists, however, for the chemical compositions and crystal chemistry of the principal secondary minerals. Here, EDS analyses of selected secondary minerals are used to calculate the crystal chemistry of phillipsite (Table S3), analcime (Table S4), Al-tobermorite (Table S5), sulphates (Table S6) and calcite (Table S7). Table 3 summarizes the calculated formulas of the analysed zeolites.

**Table 3:** Average chemical formulae of the investigated zeolites.

STC	Zeolite	Calculated chemical formula	Zone	Ra	Rr	DEC	SEC
PHI	Phillipsite	(K_2.57_ Ca_2.11_ Na_1.26_ Mg_0.03_Ba_0.03_) (Si_10.11_ Al_5.89_)O_32_·11.33H_2_O	1	0.63	0.62–0.64	K	Ca, Na, Mg, Ba
PHI	Phillipsite	(Ca_2.67_ Na_1.60_ K_1.48_ Mg_0.22_Ba_0.03_) (Si_10.12_ Al_5.88_)O_32_·8.61H_2_O	1	0.63	0.63–0.64	Ca	Na, K, Mg, Ba
PHI	Phillipsite	(K_2.48_ Ca_1.77_ Na_1.73_ Mg_0.02_Ba_0.01_) (Si_10.36_ Al_5.64_)O_32_·7.98H_2_O	3	0.65	0.64–0.66	K	Na, Ca, Mg, Ba
PHI	Phillipsite	(K_2.36_ Ca_2.02_ Na_1.58_ Ba_0.03_Mg_0.01_) (Si_10.14_ Al_5.86_)O_32_·11.34H_2_O	4	0.63	0.62–0.64	K	Ca, Na, Ba, Mg
ANA	Analcime	Na_14.84_Ca_1.03_Mg_0.07_K_0.05_Ba_0.01_ (Al_16.63_Si_31.37_) O_96_ ·19.56H_2_O		0.65	0.64–0.67	Na	Ca, Mg, K, Ba

Parameters *STC* structure type code, *Ra* average Si/(Si + Al), *Rr* range of Si/(Si + Al), *DEC* dominant extra-framework cations, *SEC* subordinate extra-framework cations as calculated by SEM–EDS analyses (see Supplementary Information).

**Figure 9:**
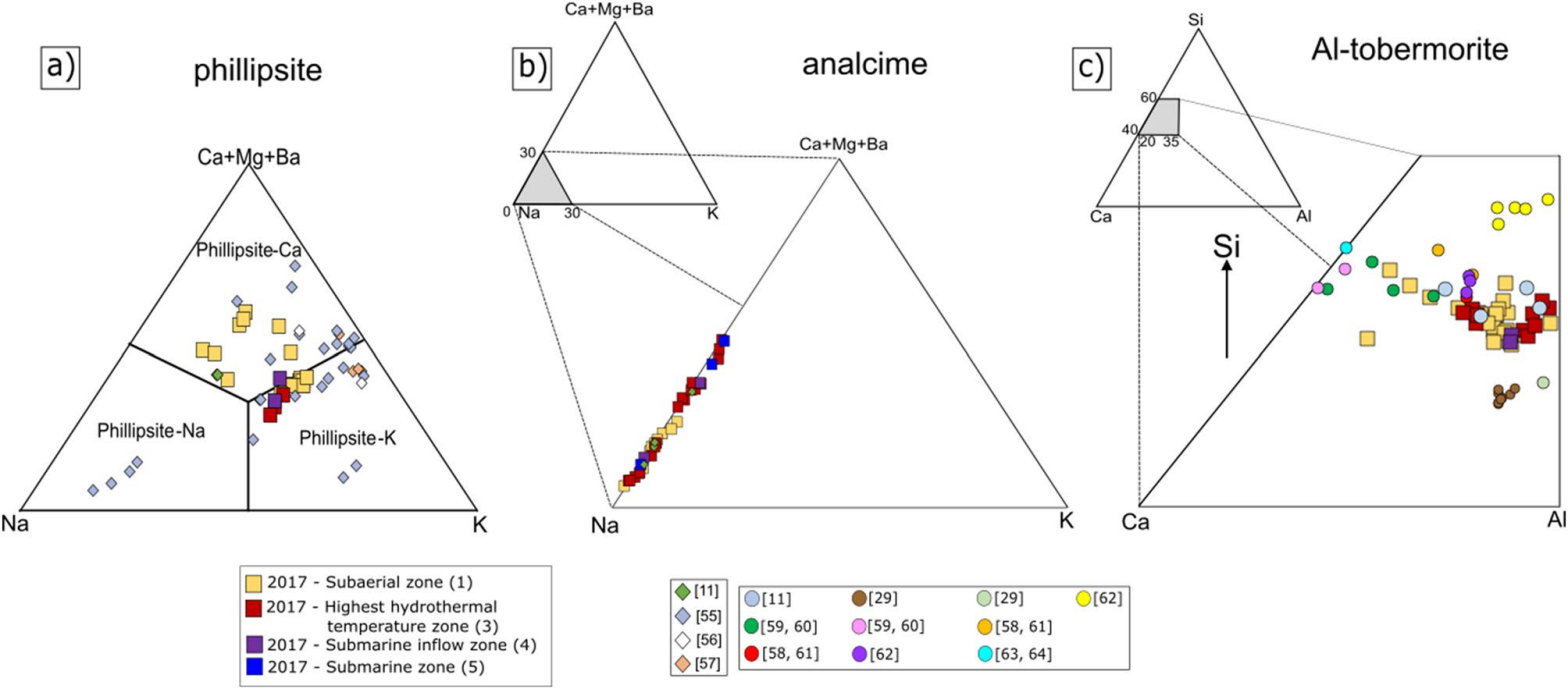
(Ca + Mg + Ba)–Na–K ternary plot of exchangeable cation content for **(a)** phillipsite and **(b)** analcime (squares). **(c)** Ca–Al–Si ternary plot for Al–tobermorite (squares). Data are plotted as a function of the structural and hydrothermal zones for Surtur crater (see Table 1, Fig. 2). Literature data for phillipsite (diamonds, panel **(a)**), analcime (small diamonds, panel **(b)**) and Al–tobermorite (circles, panel **(c)**) have been plotted for comparison (numbers in brackets show references).

Phillipsite (ideal formula: K_2_(Na,Ca_0.5_)_3_[Al_5_Si_11_O_32_]·12H_2_O; Table S3) shows the following average chemical compositions:

- (K_2.57_ Ca_2.11_ Na_1.26_ Mg_0.03_Ba_0.03_) (Si_10.11_ Al_5.89_)O_32_·11.33H_2_O for zone 1,
- (K_2.48_ Na_1.79_ Ca_1.71_ Mg_0.02_Ba_0.01_) (Si_10.40_ Al_5.60_)O_32_·7.37H_2_O for zone 3, and
- (K_2.36_ Ca_2.02_ Na_1.58_ Ba_0.03_Mg_0.01_) (Si_10.14_ Al_5.86_)O_32_·11.34H_2_O for zone 4,

in which, among the dominant extra-framework cations, K, Ca and Na^49^ (Fig. 9a), K (atoms per formula unit—a.p.f.u.) can be regarded as the prevalent element. However, a tendency towards calcium-enriched compositions exist for the phillipsite analyses in zone 1, the subaerial tuff cone (Fig. 9a, yellow squares), in which calcium is the dominant extra-framework cation:

- (Ca_2.67_ Na_1.60_ K_1.48_ Mg_0.22_Ba_0.03_) (Si_10.12_ Al_5.88_)O_32_·8.61H_2_O.

Analcime (ideal formula: Na_16_[Al_16_Si_32_O_96_)·16H_2_O; Table S4) shows an overall sodic character, with the following average chemical composition:

- Na_14.85_ Ca_1.02_ Mg_0.08_ K_0.05_ Ba_0.02_ (Al_16.62_Si_31.38_)O_96_·19.76H_2_O.

Only small differences in composition occur among the hydrothermal zones (Fig. 9b). As expected, the extra-framework site is occupied by Na^49^, with minor Ca (1.02 a.p.f.u.) and very low K (0.05 a.p.f.u.) and Ba (0.02 a.p.f.u.).

Al-tobermorite, a tobermorite crystal with 11 Å interlayer spacing that contains Al^3+^ substitution at the Si^4+^ tetrahedral site, has the generalized formula: [Ca_4_(Si_5.5_Al_0.5_O_17_H_2_)]Ca_0.2_·Na_0.1_·4H_2_O]^50^ (Table S5). The crystal chemistry measurements and calculations, plotted in the Ca–Al–Si ternary diagram of Fig. 9c, show a trend towards increasingly Al-rich compositions with temperature. The crystals in the subaerial tuff cone generally contain less alumina (Fig. 9c, yellow squares) and those crystals in the highest temperature submarine deposits display a range of more alumina-enriched compositions (Fig. 9c, red squares). Overall, the crystals have the following average chemical composition:

- Ca_4.17_ Al_0.72_ Si_6.00_ Na_0.06_ K_0.05_(O,OH)_18_·6.18H_2_O.

Finally, EDS data from sulphate-enriched microstructures (Table S6) confirm the presence of gypsum, with an H_2_O content between 2.84 and 23.10 wt%. Gypsum (ideal formula: CaSO_4_·2H_2_O) has the following average chemical composition:

- Ca_1.98_S_2.02_O_4_·1.71H_2_O.

### Activity diagrams

**Figure 10:**
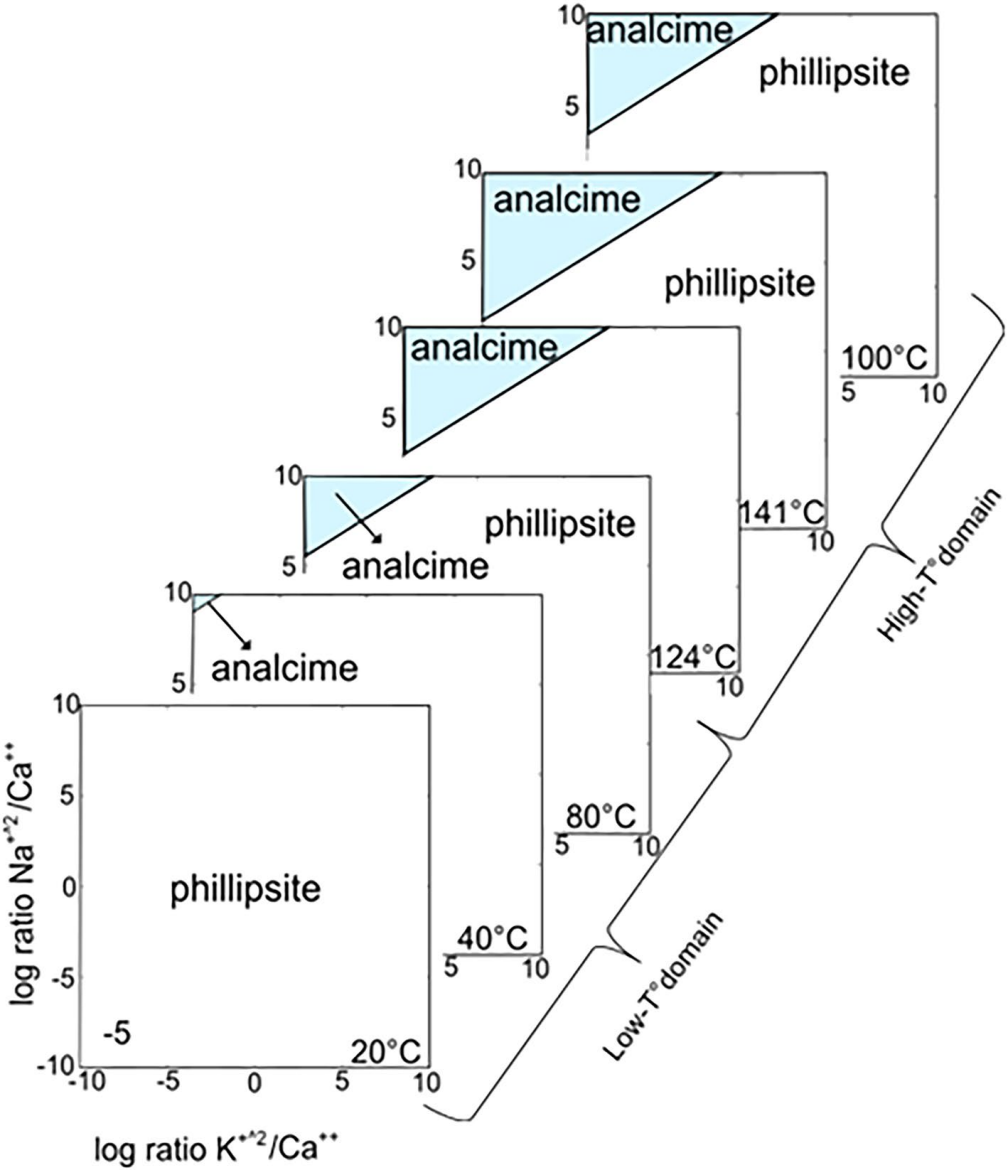
Log a K^+^ 2/Ca^++^ vs. Log a Na^+^ 2/Ca^++^ equilibrium activity diagram for phillipsite and analcime calculated for low-temperature and high-temperature domains using thermodynamic data consistent with the Surtur system (Table 3). The maximum temperatures measured are 141 °C in 1980 and 124 °C in 2017 at approximately 100 m b.s. (Fig. 2)^10,11,14,15^.

Thermodynamic calculations were performed to evaluate the stability fields of the zeolite phases, analcime and phillipsite, along the SE-02b borehole, by implementing an internal GWB database^43^ with free Gibbs energy, and calculated by an ACT2 module for different temperature values corresponding to the thermal domains defined by borehole temperature measurements. The model has predicted stability fields for zeolites from diverse geologic environments (e.g. Yucca Mountain Tuff^1,51^, Campanian Ignimbrite^52^, and Sabatini Volcanic District Yellow Tuff^53^). Following^36^, the simulation accounts for a water activity fixed at 1, with the variation of temperature. The phase diagrams (Fig. 10) predict the presence of phillipsite and analcime for the selected thermodynamic conditions, temperature, cation and silica activity. The results are quite consistent with the Surtur system. Notably, the analcime stability field systematically increases with increasing temperature from 40 to 141 °C, whereas the phillipsite field decreases with increasing temperature.

## Discussion

Progressive hydrothermal alteration within the subaerial and submarine hydrothermal system is recorded by two principal processes: (i) alteration of fresh basaltic glass and primary minerals, mainly olivine and plagioclase, and (ii) precipitation of zeolites and other secondary mineral phases, which fill vesicles in pyroclasts and pore space in the ash matrix of the lapilli tuff. Fresh basaltic glass experiences alteration which is extensive in many deposits but nonetheless significant variations in the secondary mineralogical assemblages can be recognised (Figs. 3, 4, 5, 6, 7).

The results of the chemical analyses of apparent sideromelane and newly formed phases (Figs. 8, 9) and the thermodynamic modelling of zeolite stability fields (Fig. 10) indicate two generalized temperature and alteration domains in the Surtur deposits. A low-temperature domain (T < 90–100 °C, measured in 2017) corresponds to the subaerial and the lowermost submarine deposits, at 0–58 and 140–180 m b.s. Here some apparent sideromelane in pyroclasts containing fresh olivine and higher plagioclase concentrations is preserved. A high-temperature domain (T > 100–124 °C, measured in 2017), corresponds to the upper submarine deposits, located at 50–140 m b.s. Here basaltic glass is almost completely altered. At 95–110 m b.s. the maximum borehole temperature, 141 °C measured in 1980, had decreased to 124 °C in 2019 (Fig. 2). The overall extent of alteration likely results from different thermal histories, as well as variations in fluid compositions^20^. However, some apparent sideromelane is occasionally preserved in samples from the high-temperature domain where alteration processes are observed to be at an advanced stage. Various studies have shown that the rates of alteration are dependent, in part, on temperature (e.g.^17^). However, our results indicate that the microenvironment (i.e., fine-scale conditions related to direct glass-fluid interaction) may be just as influential as macroenvironment (i.e., physical parameters of the system, such as temperature) in controlling the development of authigenic alteration products and the overall consolidation and lithification of tephra.

The integrated results of numerous petrographic, SEM and X-ray microdiffraction-microfluorescence analyses of Surtur lapilli tuff^11,13,17,21^ reveal that samples possess particular microstructural environments, with specific fluid–glass–crystal interactions, such that the fluid compositions in vesicles (or pores) change over time to precipitate crystals with evolving compositions. These compositions range from analcime (sodic) and phillipsite (calcic or potassic) to Al–tobermorite (more or less rich in aluminium) (Table 3). In sample RS-8 (92.6 m b.s.), for example, the microenvironment of glass alteration (Fig. 5d) is very different from that of the altered olivine crystal (Fig. 5f) and, also, from the microenvironment in sample RS-9 (101.5 m b.s.; Fig. 6f), where gypsum crystallizes. Yet the temperature history is the same for both the samples (T > 100–124 °C).

**Figure 11:**
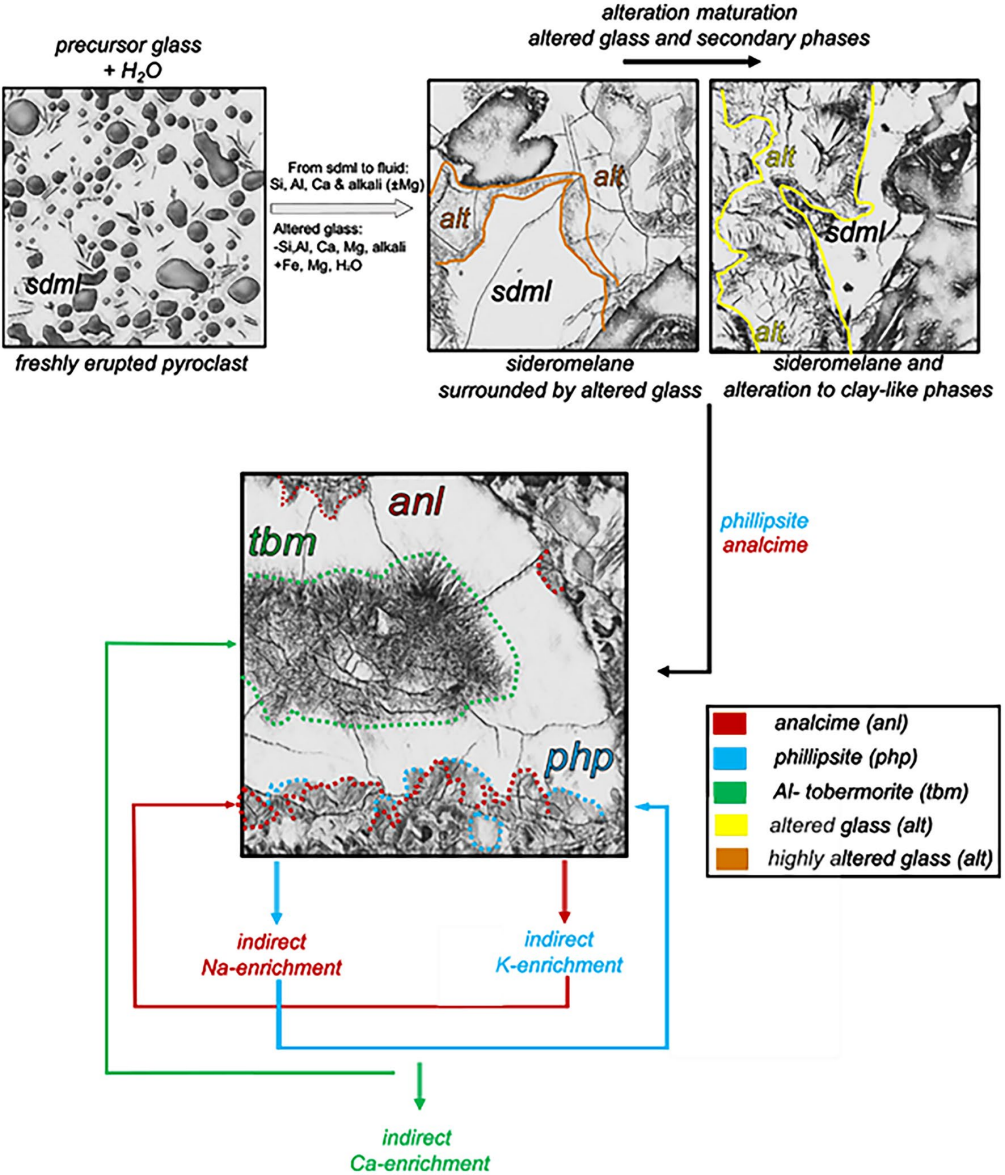
Representation of glass alteration and secondary phase formation based on the microstructural features of RS-1, RS-2 and RS-8. Elemental gains and losses are based on SEM-EDS chemical data from microstructures (see Supplementary Material).

The mineralogical progressions of secondary phases in the Surtur deposits are linked to the cumulative effect of fluid-rock interactions, measured through borehole fluid compositions^20^. The first component to be affected is basaltic glass (Figs. 3,5), which changes composition during alteration (Fig. 11).

Glass alteration supplies mobile chemical components to aqueous solutions, which then precipitate as secondary phases in vesicles and pores of the lapilli tuff during the evolution of the hydrothermal system^54^.

• Elements deriving from primary minerals are also transported in solution, and eventually consumed to form secondary minerals.

• Hydration and the associated breakdown of glass and primary minerals remove Na, K, Ca, Si, Al, and to a lesser extent Mg, which are transferred to fluids. The starting materials are then enriched in Fe, H_2_O, and overall in Mg, and progressively depleted in Si, Al, Na, K and Ca.

• Only a few EDS analyses of altered glass from zones 1 and 4 contain Mg, suggesting limited leaching of Mg from basaltic glass and primary crystals during early stages of alteration, which is consistent with the relatively low degree of alteration^21^.

Incipient alteration is characterized by thin rinds around the unaltered glassy pyroclasts and the precipitation of secondary phases (phillipsite, analcime and Al-tobermorite) as surface coating in vesicles. Alteration proceeds with the formation of poorly-ordered smectite-like clays, the low-Ca and Ti-poor products of authigenic alteration (Fig. 8), and the progressive pervasive precipitation of secondary phases. A strong immobility of Ti and Fe (Fig. 8b,e) occurs in the highly altered glass, indicating that alteration has progressed so far as to produce clay minerals.

The type of secondary phase depends on the chemical components supplied to the aqueous solution. Phillipsite and analcime appear to be early alteration products in the crystallization sequence. They can form contemporaneously, possibly in response to the K/Na ratio of the aqueous solution, thus leading to the indirect enrichment in K or Na and the formation of one or the other phase. This is consistent with petrographic and SEM observations of the spatial distribution of secondary minerals: from early to late formation and from outermost to innermost portions of cavities, phillipsite-analcime and Al-tobermorite occur sequentially (Figs. 4c,d, 5e, 6c–e).

**Figure 12:**
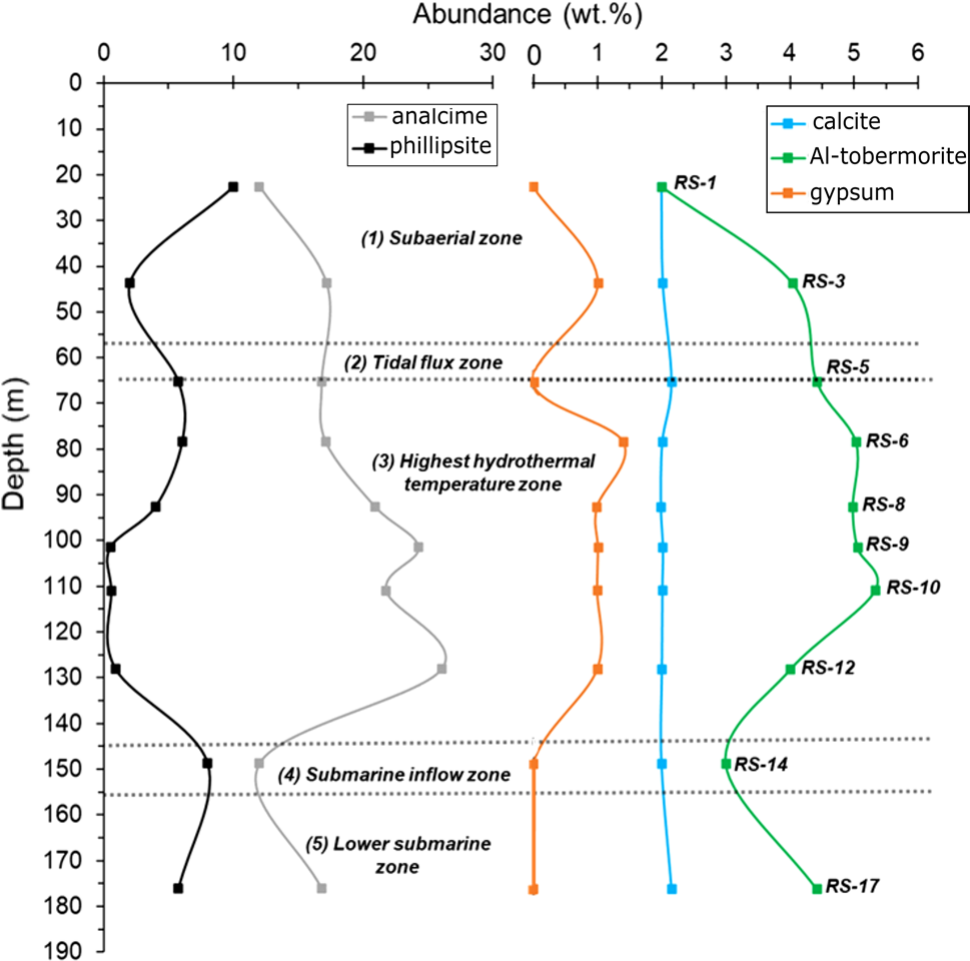
Distribution of phillipsite, analcime, Al-tobermorite, calcite and gypsum in samples from SE-02b drill core based on quantitative X-ray diffraction analysis. Note the negative correlation between phillipsite and analcime concentrations. See Fig. 2 for temperature profiles.

For glass alteration, however, temperature also plays a role^11,28,65,66^. Crystallization of phillipsite, analcime and Al–tobermorite positively correlates with temperature, but phillipsite and analcime correlate negatively with each other. The activity diagram shows that the phillipsite field progressively narrows with increasing temperature, at the expenses of the analcime stability field (Fig. 10). Thus, at higher temperatures, the more favourable crystallization of analcime strongly influences the presence of phillipsite. QXRPD analyses (Fig. 12, Table 2) indicate that phillipsite content reaches 10 wt% in samples from subaerial zone 1, decreases to 1 wt% in the highest temperature zone 3, and increases to 8 wt% in the lower temperature submarine inflow zone 4. By contrast, analcime content occurs at 12–17 wt% in zones 1, 2, 4 and 5, yet reaches 26 wt% in zone 3.

Al-tobermorite content is 2–4 wt% in zones 1, 2, 4 and 5, reaching 5 wt% in zone 3, and 3–4 wt% in zones 4 and 5, thus showing only a slight increase at higher temperature.

The compositions of phillipsite (Fig. 9a) are similar to phillipsites occurring in basaltic rocks^55–57^, although some samples from the subaerial zone 1, above water level, are more Ca-rich. This could perhaps be related to the influence of fluids of meteoric origin and/or to calcium derived from the dissolution of glass (Table S1). The possible concomitant occurrence of similar species with a different crystalline structure (e.g., merlinoite) requires further investigations.

The reflections at 1.54 Å resemble those of trioctahedral clay minerals, such as saponite; however, dioctahedral clay minerals with 1.51 Å reflections, such as nontronite, were reported, together with saponite for the SE-01 drill core^11,13^. Future investigations on clay-size fractions are needed to precisely identify the types and percentages of clay minerals.

## Conclusions

Systematic analyses of apparent sideromelane, altered glass and secondary minerals in the microstructures of 50-year old pyroclastic deposits at Surtsey volcano record compositional variations that provide new insights into processes that produce in situ mineral growth in young oceanic basalt. The subaerial and submarine lapilli tuff samples are distributed along a 192 m, vertical drill core, SE-02b, acquired in 2017 through the Surtur structure.

A database of SEM–EDS data, provides a crystal chemical characterization of the secondary phases. With these data, the distribution of the exchangeable ion content in an analcime–phillipsite–Al–tobermorite mineral system is evaluated in five hydrothermal zones within the subaerial and submarine lapilli tuff deposits. The compositional analyses also evaluate chemical changes in altered glass through the hydrothermal zones.

The integrated analyses reveal:

i. Multi-stage alteration of basaltic glass and precipitation of secondary minerals from interstitial solutions as pore space cement.
ii. Large amorphous content (53–60 wt%) in many lapilli tuff samples shown by QXRPD analyses. This includes a small amount of apparently fresh glass that may persist in some deposits as well as probable nanocrystalline phases with low long-range order^13^ and/or poorly-ordered phases, such as smectite-like clay mineral(s). SEM–EDS analyses describe chemical variations among these materials and offer a means to decipher the complexity of phases typically described as sideromelane and palagonitized glass.
iii. Variable compositions and exchangeable cation content of secondary minerals, pointed out by SEM–EDS analyses. For example, phillipsite has calcium-enriched compositions in the subaerial tuff cone; there is a trend towards increasingly Al-rich Al-tobermorite compositions with temperature. Analcime, however, has a consistently sodic character.
iv. Systematic increase of the analcime stability field with increasing temperature (40–141 °C) shown in activity diagrams. By contrast, the phillipsite stability field decreases with increasing temperature.

The complex history of secondary mineral precipitation in pore and vesicle microstructures indicates an important chronological evolution of pore fluids at the millimeter-to-meter scale of the lapilli tuff. This suggests that interaction of pore fluids with changing reactive components in microenvironments could be as just as influential as the temperature in determining the reaction pathways that produce subsequent secondary phases.

Descriptions of the finely resolved, spatial frameworks of mineralization in young, active and well-monitored hydrothermal system of Surtsey volcano offer new insights into the structural organization of alteration process and secondary mineral growth in oceanic basalts. The innovative analytical approach taken in this research will provide a valuable template for further mineralogical and crystal chemical explorations of these processes in modern and ancient basaltic environments.

### Supplementary Information

Supplementary Figure S1.

Supplementary Figure S2.

Supplementary Figure S3.

Supplementary Table S1.

Supplementary Table S2.

Supplementary Table S3.

Supplementary Table S4.

Supplementary Table S5.

Supplementary Table S6.

Supplementary Table S7.

## Data Availability

All data generated or analysed during this study are included in this published article (and its Supplementary Information files).
