# Supplementary material for: Authigenic mineralization in Surtsey basaltic tuff deposits at 50 years after eruption
Source: Sci Rep. 2023 Dec 21;13:22855. doi: 10.1038/s41598-023-47439-4 (PMC10739796; doi:10.1038/s41598-023-47439-4)
Supplement: Supplementary file 1 — Supplementary Figure S1. [file 41598_2023_47439_MOESM1_ESM.pdf]

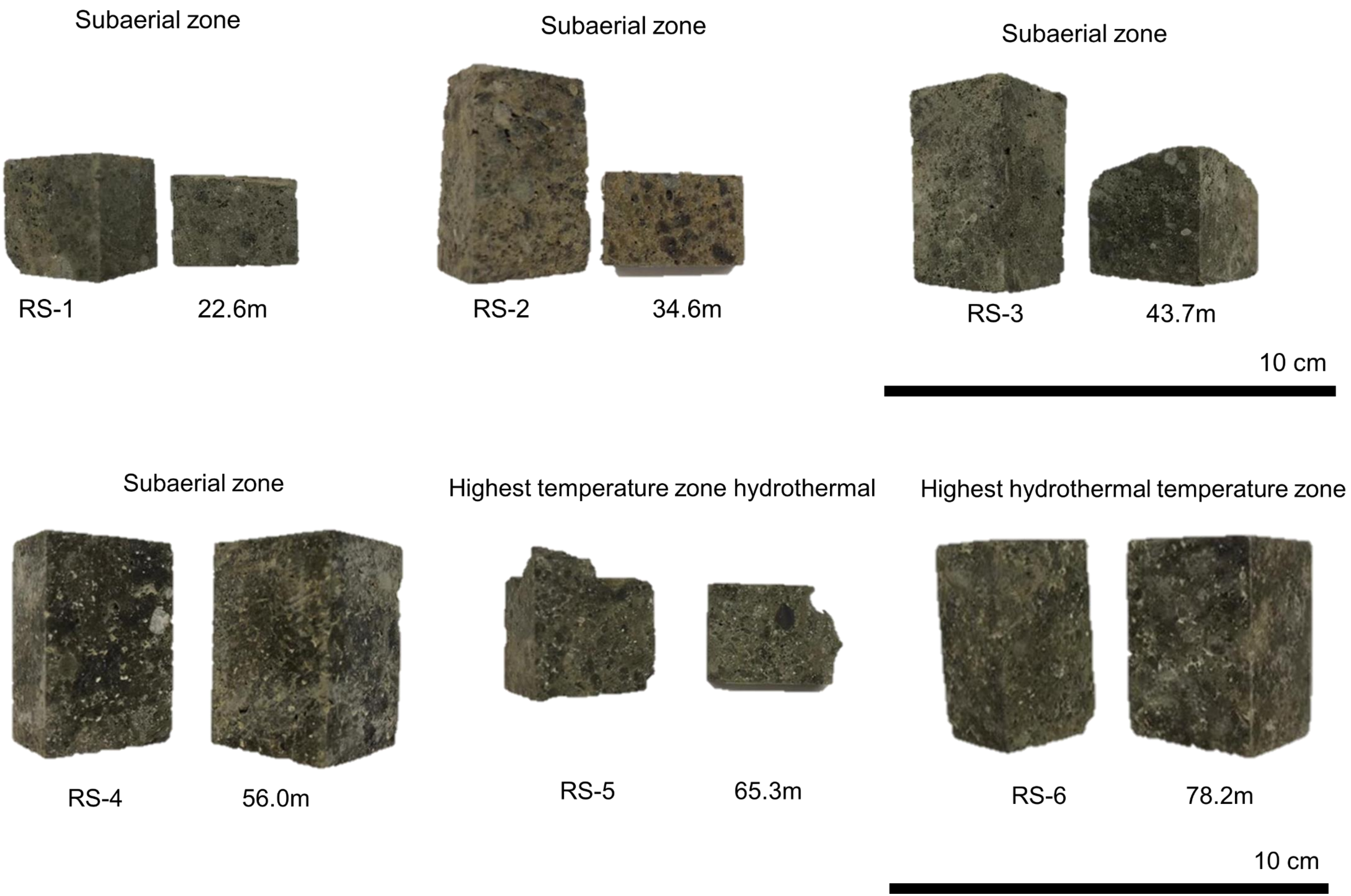
Subaerial zone
RS-1
22.6m
Subaerial zone
RS-2
34.6m
Subaerial zone
RS-3
43.7m
10 cm
Subaerial zone
RS-4
56.0m
Highest temperature zone hydrothermal
RS-5
65.3m
Highest hydrothermal temperature zone
RS-6
78.2m
10 cm

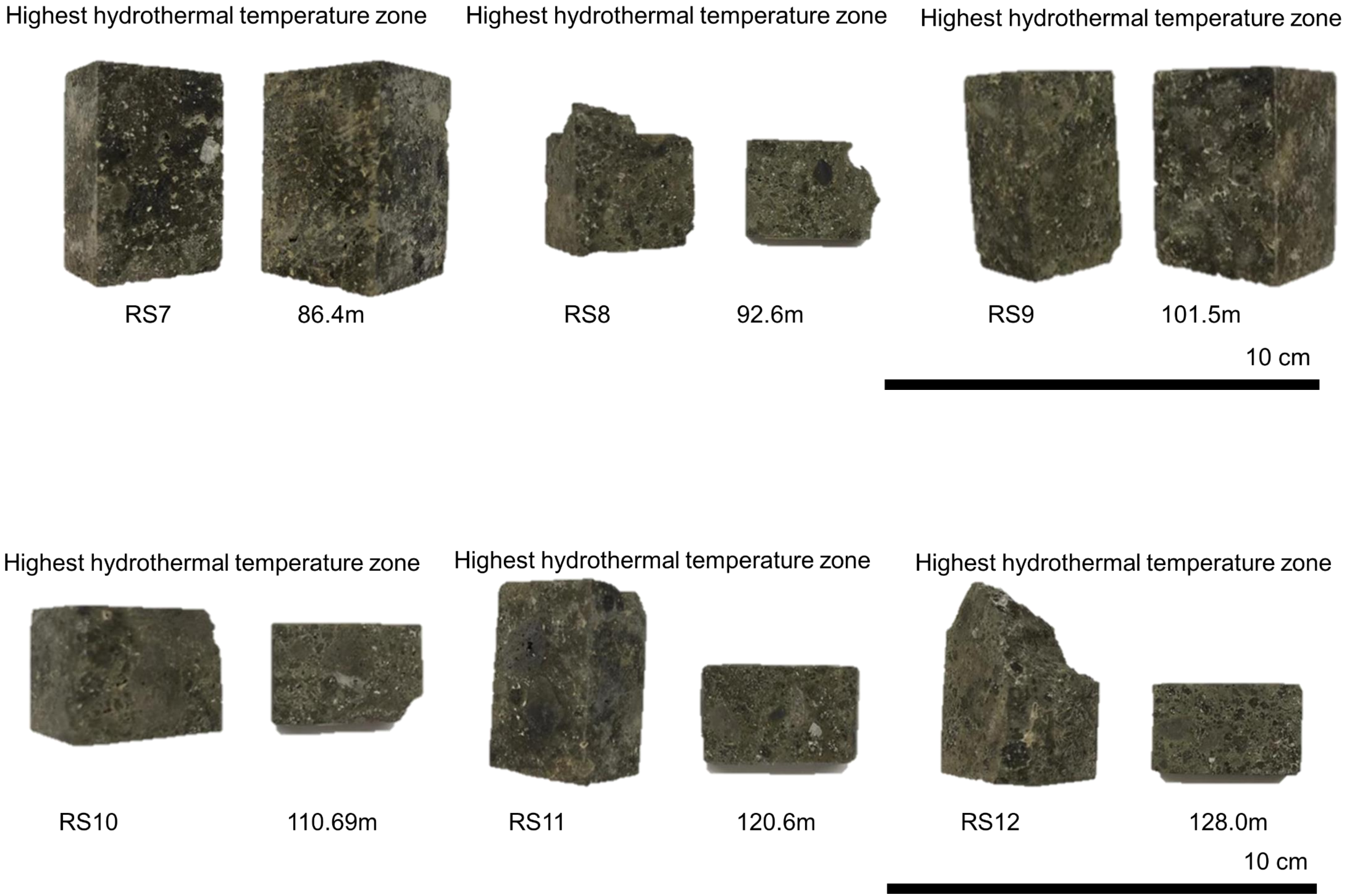

Highest hydrothermal temperature zone
Highest hydrothermal temperature zone
Highest hydrothermal temperature zone
RS7
86.4m
RS8
92.6m
RS9
101.5m
10 cm
Highest hydrothermal temperature zone
Highest hydrothermal temperature zone
Highest hydrothermal temperature zone
RS10
110.69m
RS11
120.6m
RS12
128.0m
10 cm

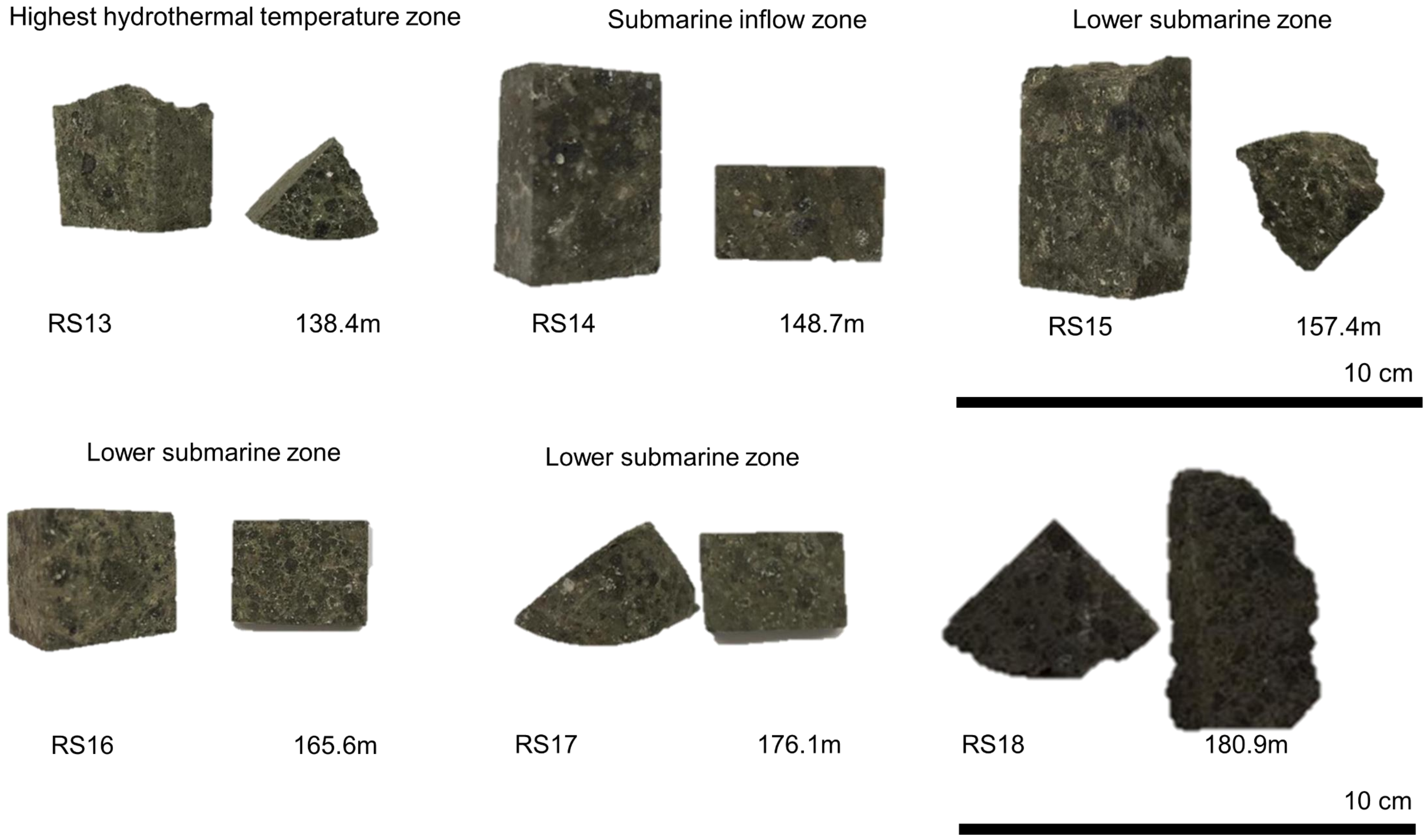

Supplementary Figure S1. Reference samples (RS) of lapilli tuff from SE-02b vertical borehole.
m=meters below surface
