# Supplementary figures and images for: Authigenic mineralization in Surtsey basaltic tuff deposits at 50 years after eruption
Source: Sci Rep. 2023 Dec 21;13:22855. doi: 10.1038/s41598-023-47439-4 (PMC10739796; doi:10.1038/s41598-023-47439-4)

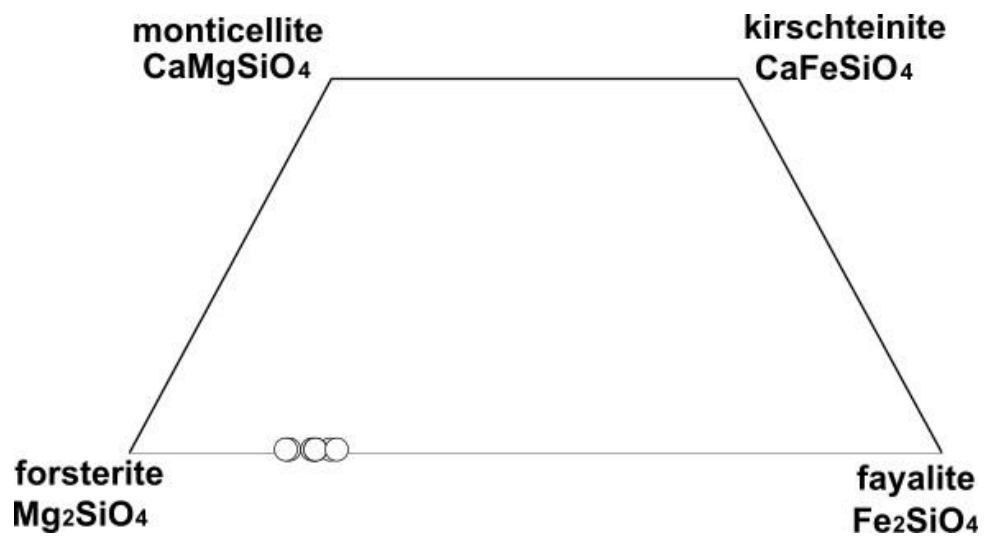

Supplementary Figure S2. Composition of the analyzed olivine crystals (HOLE C).

Supplement: Supplementary file 2 — Supplementary Figure S2. [file 41598_2023_47439_MOESM2_ESM.pdf]

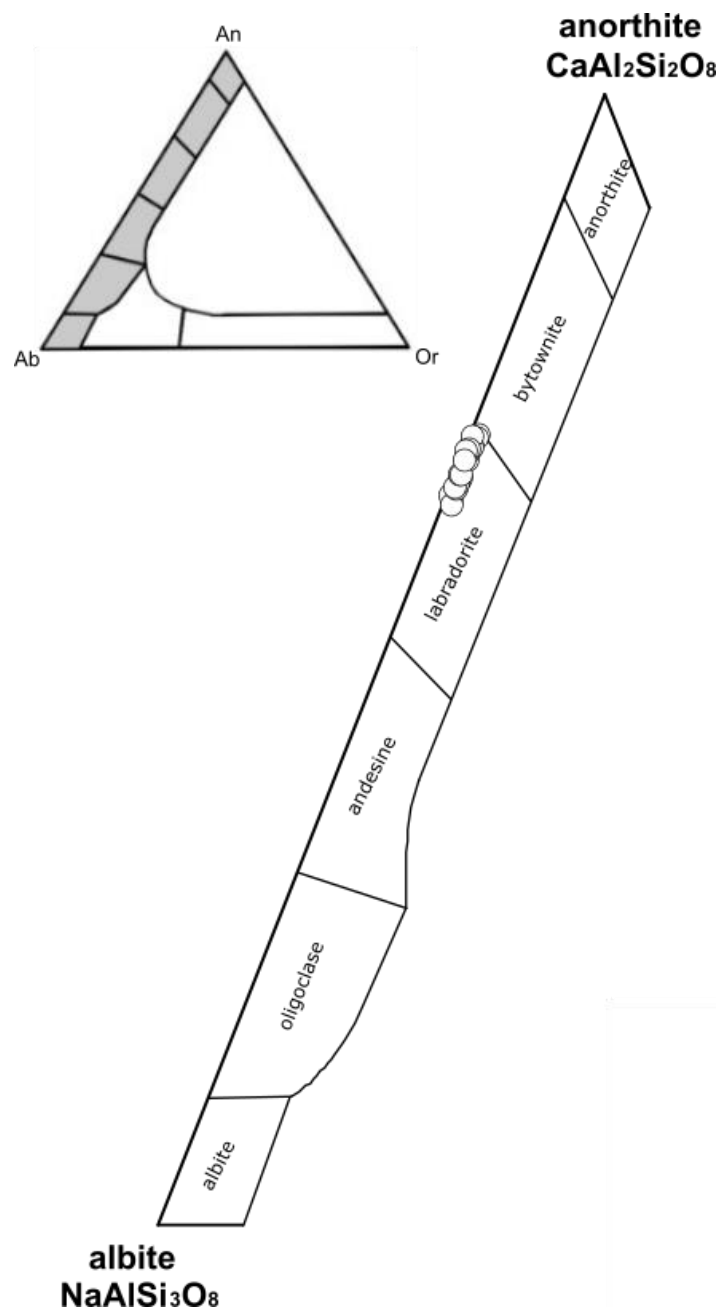

Supplementary Figure S3. Composition of the analyzed plagioclase crystals (HOLE C).

Supplement: Supplementary file 3 — Supplementary Figure S3. [file 41598_2023_47439_MOESM3_ESM.pdf]
