# Supplementary material for: Authigenic mineralization in Surtsey basaltic tuff deposits at 50 years after eruption
Source: Sci Rep. 2023 Dec 21;13:22855. doi: 10.1038/s41598-023-47439-4 (PMC10739796; doi:10.1038/s41598-023-47439-4)
Supplement: Supplementary file 4 — Supplementary Table S1. [file 41598_2023_47439_MOESM4_ESM.pdf]

S4. Major elements (in wt.%, normalized to 100 water and volatile-free) for the analyzed sideromelane.

	HOLE-C													
Sample	RS-2	RS-2	RS-2	RS-2	RS-2	RS-2	RS-3	RS-14	RS-14	RS-14	RS-18	RS-18	RS-18	RS-18
SiO_2	47.20	47.16	47.16	46.89	47.49	47.04	47.63	46.78	46.92	46.78	46.40	46.87	46.69	46.51
TiO_2	2.86	2.66	2.64	2.53	2.67	2.50	2.47	2.78	2.94	2.67	3.20	2.77	2.75	3.01
Al_2O_3	16.28	16.45	16.24	16.82	16.52	16.60	16.50	16.52	16.72	16.87	16.60	16.16	16.57	16.35
FeO**	12.55	12.32	12.45	12.42	12.60	12.68	11.93	12.89	12.45	12.51	13.38	13.48	13.10	13.40
MnO	0.34	0.25	0.38	0.27	0.26	0.13	0.15	0.47	0.37	0.16	0.15	0.08	0.32	0.36
MgO	5.94	6.01	6.28	6.25	6.03	6.22	6.12	5.48	5.68	6.29	5.62	5.73	5.70	5.63
CaO	9.80	9.85	9.83	9.91	9.82	10.02	10.03	9.55	9.38	9.83	9.30	9.48	9.46	9.37
Na_2O	3.93	4.06	4.06	3.93	3.46	3.87	4.09	4.29	4.28	3.98	4.13	4.15	4.22	4.12
K_2O	0.70	0.75	0.75	0.67	0.71	0.68	0.63	0.83	0.89	0.69	0.86	0.85	0.77	0.77
P_2O_5	0.39	0.49	0.20	0.30	0.44	0.27	0.46	0.41	0.37	0.22	0.35	0.42	0.41	0.48
Total	100.00	100.00	100.00	100.00	100.00	100.00	100.00	100.00	100.00	100.00	100.00	100.00	100.00	100.00

** total Fe expressed as FeO
