# Supplementary material for: Authigenic mineralization in Surtsey basaltic tuff deposits at 50 years after eruption
Source: Sci Rep. 2023 Dec 21;13:22855. doi: 10.1038/s41598-023-47439-4 (PMC10739796; doi:10.1038/s41598-023-47439-4)
Supplement: Supplementary file 5 — Supplementary Table S2. [file 41598_2023_47439_MOESM5_ESM.pdf]

S5. Major elements (wt.%) for the analyzed altered glass.

Sample	RS-2	RS-2	RS-2	RS-2	RS-2	RS-2	RS-2	RS-3	RS-3	RS-3	RS-3	RS-3	RS-3
SiO_2	32.52	33.42	34.46	34.19	34.00	34.36	35.01	36.03	36.36	32.90	34.41	36.45	35.09
TiO_2	4.48	4.25	3.85	3.71	3.91	6.20	3.84	3.98	3.44	4.06	3.69	3.55	3.79
Al_2O_3	10.82	11.66	12.30	13.16	12.36	10.59	9.94	10.32	10.84	10.25	10.52	11.07	10.50
FeO**	20.26	18.78	18.13	17.02	18.48	19.49	17.23	15.71	15.74	17.49	16.88	16.92	16.83
MnO	0.29	0.30	0.14	0.26	0.20	0.32	0.31	0.29	0.30	0.16	0.34	0.31	0.40
MgO	2.10	4.65	3.34	3.48	3.01	8.92	7.92	3.81	4.65	7.05	5.41	5.64	5.41
CaO	9.00	7.53	9.14	9.14	8.83	5.90	7.88	9.16	8.29	7.61	8.18	8.58	8.03
Na_2O	0.65	0.55	0.76	0.90	0.59	0.51	0.48	0.65	0.64	0.57	0.49	0.55	0.57
K_2O	0.51	0.59	0.46	0.60	0.57	0.49	0.77	0.48	0.44	0.36	0.39	0.43	0.37
P_2O_5	0.60	0.07	0.48	0.58	0.51	0.52	0.67	0.68	0.15	0.76	0.57	0.50	0.49
SO_3	0.23	0.06	0.12	-	0.02	0.12	0.18	0.22	0.08	0.03	0.25	0.14	0.17
Cl^-	0.13	0.14	0.17	0.17	0.17	0.04	0.05	0.02	-	0.05	-	0.05	0.09
Total	81.59	82.23	83.45	83.45	82.82	87.91	84.47	81.55	81.05	81.62	81.35	85.06	81.77
H_2O	18.41	17.77	16.55	16.55	17.18	12.09	15.53	18.45	18.95	18.38	18.65	14.94	18.23

S5. (continue)

	HOLE - C														
Sample	RS-4	RS-4	RS-8	RS-8	RS-8	RS-8	RS-8	RS-8	RS-9	RS-9	RS-9	RS-9	RS-9	RS-14	RS-14
SiO_2	35.75	34.65	34.45	32.99	33.55	38.12	35.03	36.18	34.09	32.13	38.20	41.01	36.88	33.32	35.96
TiO_2	3.65	3.77	2.40	3.99	4.09	0.14	0.11	1.95	5.09	3.84	-	0.07	-	4.14	4.02
Al_2O_3	12.91	11.41	11.09	10.85	12.33	8.78	10.19	11.53	11.00	10.16	9.29	9.39	10.39	10.97	10.23
FeO**	17.40	17.22	18.69	17.95	19.53	25.69	25.77	21.20	18.60	17.98	26.15	17.47	26.71	18.99	18.91
MnO	0.23	0.39	0.48	0.24	0.30	0.13	0.22	0.39	0.24	0.26	0.12	0.11	0.34	0.21	0.10
MgO	4.57	6.56	10.00	9.33	9.93	10.72	10.75	11.75	9.21	9.77	11.65	15.15	12.41	2.67	6.71
CaO	8.64	8.99	6.01	7.23	6.54	3.14	2.44	4.23	8.98	6.87	2.63	2.26	2.67	8.50	7.78
Na_2O	0.67	0.69	0.45	0.44	0.42	0.49	0.43	0.41	0.52	0.55	0.47	0.34	0.30	0.82	0.72
K_2O	0.59	0.53	1.19	0.43	0.50	0.33	0.65	0.50	0.26	0.33	0.20	0.18	0.27	0.51	0.33
P_2O_5	0.44	0.51	0.83	0.42	0.33	-	-	-	0.50	0.64	0.06	0.11	0.00	0.61	0.41
SO_3	0.07	0.18	0.20	0.18	-	-	-	-	0.24	0.06	0.05	-	0.07	-	-
Cl^-	0.30	0.18	0.27	0.14	0.17	0.11	0.08	0.19	0.15	0.11	0.04	0.11	0.07	0.14	0.12
Total	83.53	85.43	86.39	84.42	88.14	87.66	85.82	88.57	89.33	82.96	88.90	8626	90.13	81.09	85.43
H_2O*	14.47	14.57	13.61	15.58	11.86	12.34	14.18	11.43	10.67	17.04	11.10	13.74	9.87	18.91	14.57

*calculated by difference; ** total Fe expressed as FeO
