# Supplementary material for: Authigenic mineralization in Surtsey basaltic tuff deposits at 50 years after eruption
Source: Sci Rep. 2023 Dec 21;13:22855. doi: 10.1038/s41598-023-47439-4 (PMC10739796; doi:10.1038/s41598-023-47439-4)
Supplement: Supplementary file 6 — Supplementary Table S3. [file 41598_2023_47439_MOESM6_ESM.pdf]

S6. Chemical analyses for the investigated phillipsite.

Sample	RS-2	RS-2	RS-2	RS-2	RS-2	RS-2	RS-2	RS-2	RS-3	RS-3	RS-3	RS-3	RS-3	RS-3	RS-3	RS-3	RS-3	RS-3	RS-4	RS-4	RS-4
SiO_2	49.01	47.68	50.39	53.41	50.2	51.17	50.11	52.42	47.87	46.75	47.8	47.47	51.85	47.75	47.07	47.77	48.09	47.81	49.39	49.87	49.29
TiO_2	0.05	0.18	0.45	0.20	0.03	0.08	0.29	0.11	-	-	0.18	-	0.06	-	-	0.04	0.06	-	-	0.12	-
Al_2O_3	23.81	23.35	24.62	25.82	25.27	25.11	25.2	26.08	23.54	23.5	23.79	23.18	25.16	24.02	23.62	24.64	24	23.56	23.8	23.9	24.1
FeO_{tot}	0.49	0.25	0.39	0.64	0.4	0.34	0.35	0.4	-	-	-	0.02	0.25	0.04	0.11	0.1	0.44	0.03	0.19	0.46	-
MnO	0.04	0.1	0.01	0.02	-	-	-	-	0.04	0.11	-	-	0.04	-	-	-	0.15	-	-	0.09	0.02
MgO	0.07	0.7	0.22	1.89	0.21	0.06	0.31	0.44	-	0.07	0.08	0.04	0.11	0.06	0.08	0.02	0.19	0.02	0.13	0.15	0.02
CaO	6.72	7.2	9.19	8.03	8.89	7.9	8.71	8.2	6.77	6.76	6.78	6.64	6.41	6.81	7.12	6.89	6.88	6.64	6.66	6.12	7.2
Na_2O	3.66	3.65	1.68	2.31	2.47	1.77	2.2	3.82	2.14	2.1	2.13	2.11	2.36	2.04	2.09	2.06	2.59	2.36	2.13	2.77	1.93
K_2O	4.17	2.6	4.66	3.4	3.2	5.55	3.1	3.42	7.17	6.8	7.03	6.82	6.33	7.04	6.96	7.01	6.71	6.73	7.25	6.64	7.19
BaO	0.15	0.03	0.34	0.25	0.54	0.38	0.34	0.2	0.26	0.25	0.02	0.42	0.21	0.29	0.19	0.36	0.06	0.35	0.25	0.27	0.41
Total	88.17	85.74	91.95	95.97	91.21	92.36	90.61	95.09	87.79	86.34	87.81	86.7	92.78	88.05	87.24	88.89	89.17	87.5	89.8	90.3	90.1
H_2O*	11.83	14.26	8.05	4.03	8.79	7.64	9.39	4.91	12.21	13.66	12.19	13.3	7.22	11.95	12.76	11.11	10.83	12.5	10.1	9.61	9.83
cations based on 32 oxygens																					
Si	10.17	10.09	10.06	10.10	10.04	10.16	10.05	10.05	10.12	10.04	10.07	10.15	10.25	10.06	10.02	9.97	10.02	10.12	10.1	10.2	10.1
Ti	0.01	0.03	0.07	0.03	0.00	0.01	0.04	0.02	0.00	0.00	0.03	0.00	0.01	0.00	0.00	0.01	0.01	0.00	0.00	0.02	0.00
Al	5.82	5.83	5.80	5.75	5.96	5.88	5.96	5.89	5.86	5.95	5.91	5.84	5.86	5.96	5.93	6.06	5.89	5.88	5.79	5.76	5.84
Fe	0.09	0.04	0.07	0.10	0.07	0.06	0.06	0.06	0.00	0.00	0.00	0.00	0.04	0.01	0.02	0.02	0.08	0.01	0.03	0.08	0.00
Mn	0.01	0.01	0.00	0.00	0.00	0.00	0.00	0.00	0.01	0.02	0.00	0.00	0.01	0.00	0.00	0.00	0.02	0.00	0.00	0.01	0.00
Mg	0.02	0.22	0.07	0.53	0.06	0.02	0.09	0.13	0.00	0.02	0.03	0.01	0.03	0.02	0.03	0.01	0.06	0.01	0.04	0.05	0.01
Ca	1.49	1.63	1.97	1.63	1.91	1.68	1.87	1.68	1.53	1.56	1.53	1.52	1.36	1.54	1.62	1.54	1.54	1.51	1.47	1.34	1.59
Na	1.47	1.50	0.65	0.85	0.96	0.68	0.86	1.42	0.88	0.87	0.87	0.87	0.90	0.83	0.86	0.83	1.05	0.97	0.85	1.10	0.77
K	1.10	0.70	1.19	0.82	0.82	1.41	0.79	0.84	1.93	1.86	1.89	1.86	1.60	1.89	1.89	1.87	1.78	1.82	1.91	1.73	1.89
Ba	0.01	0.00	0.03	0.02	0.04	0.03	0.03	0.02	0.02	0.02	0.00	0.04	0.02	0.02	0.02	0.03	0.00	0.03	0.02	0.02	0.03
H_2O	8.18	10.06	5.36	2.54	5.86	5.06	6.28	3.14	8.60	9.78	8.56	9.48	4.75	8.39	9.05	7.73	7.52	8.82	6.98	6.55	6.74
Total	20.19	20.06	19.89	19.82	19.85	19.92	19.75	20.11	20.35	20.34	20.32	20.29	20.06	20.32	20.39	20.33	20.44	20.33	20.3	20.3	20.2
E%	3.18	-1.95	-2.72	-4.54	2.80	6.01	5.79	-0.23	-1.13	-0.32	0.56	-0.53	10.09	1.34	-2.57	3.55	-2.94	0.16	-0.48	1.55	-1.16
CEC_{teor}	4.52	4.65	4.96	5.30	4.82	4.65	4.67	5.13	4.66	4.60	4.64	4.57	4.47	4.65	4.76	4.67	4.82	4.61	4.70	4.60	4.78
Si/Al	1.75	1.73	1.74	1.76	1.69	1.73	1.69	1.71	1.73	1.69	1.70	1.74	1.75	1.69	1.69	1.65	1.70	1.72	1.76	1.77	1.73
K/Na	0.75	0.47	1.83	0.97	0.85	2.06	0.93	0.59	2.20	2.13	2.17	2.12	1.76	2.27	2.19	2.23	1.70	1.87	2.24	1.57	2.45

*calculated by difference; ** otal Fe expressed as FeO; CECteor = theoretical Cation Exchange Capacity (expressed as meq/g)
