# Supplementary material for: Authigenic mineralization in Surtsey basaltic tuff deposits at 50 years after eruption
Source: Sci Rep. 2023 Dec 21;13:22855. doi: 10.1038/s41598-023-47439-4 (PMC10739796; doi:10.1038/s41598-023-47439-4)
Supplement: Supplementary file 7 — Supplementary Table S4. [file 41598_2023_47439_MOESM7_ESM.pdf]

S7. Chemical analyses for the investigated analcime.

Sample	RS-2	RS-2	RS-2	RS-2	RS-3	RS-3	RS-3	RS-3	RS-4	RS-4	RS-4	RS-8	RS-8
SiO_2	51.80	52.67	53.14	52.45	50.69	51.24	51.84	52.93	51.13	52.68	54.00	50.37	52.21
Al_2O_3	23.60	23.39	24.59	23.31	22.98	23.39	23.83	23.91	23.28	23.16	23.57	22.69	23.78
FeO**	0.36	0.41	0.38	0.12	0.28	0.18	0.17	0.24	0.04	0.20	0.17	0.30	0.23
MgO	0.34	0.07	-	-	0.08	0.06	-	0.14	0.13	-	-	0.04	0.03
CaO	1.15	0.71	1.47	0.78	1.01	1.11	1.43	1.12	1.39	0.59	0.44	2.06	2.20
Na_2O	11.59	12.77	12.24	12.88	12.80	12.81	13.04	13.02	12.45	12.85	13.54	11.59	12.12
K_2O	0.14	0.06	0.05	0.09	0.04	0.02	0.04	0.03	0.13	0.08	-	0.05	-
BaO	0.10	0.02	0.05	0.06	0.08	0.15	-	0.10	0.14	0.26	-	-	0.02
Total	89.08	90.10	91.92	89.69	87.96	88.96	90.35	91.39	88.69	89.82	91.72	87.10	90.59
H_2O*	10.92	9.90	8.08	10.31	12.04	11.04	9.65	8.61	11.31	10.18	8.28	12.90	9.41
						cations based on 96 oxygens							
Si	31.33	31.54	31.19	31.55	31.21	31.18	31.08	31.29	31.20	31.67	31.72	31.27	31.17
Al	16.82	16.51	17.01	16.52	16.68	16.78	16.83	16.66	16.74	16.41	16.32	16.60	16.73
Fe	0.18	0.21	0.19	0.06	0.14	0.09	0.09	0.12	0.02	0.10	0.08	0.16	0.11
Mg	0.31	0.06	0.00	0.00	0.07	0.05	0.00	0.12	0.12	0.00	0.00	0.04	0.03
Ca	0.75	0.46	0.92	0.50	0.67	0.72	0.92	0.71	0.91	0.38	0.28	1.37	1.41
Na	13.59	14.82	13.93	15.02	15.28	15.11	15.15	14.92	14.73	14.97	15.42	13.95	14.03
K	0.11	0.05	0.04	0.07	0.03	0.02	0.03	0.02	0.10	0.06	0.00	0.04	0.00
Ba	0.02	0.00	0.01	0.01	0.02	0.04	0.00	0.02	0.00	0.06	0.00	0.00	0.00
H_2O	22.02	19.76	15.81	20.68	24.72	22.40	19.29	16.97	23.01	20.40	16.22	26.70	18.73
Total	63.11	63.64	63.29	63.74	64.10	63.99	64.10	63.86	63.85	63.65	63.83	63.42	63.48
E%	6.14	3.71	7.40	2.49	-0.91	0.11	-1.10	0.01	-1.23	3.07	2.15	-1.20	-1.03
CEC_{teor}	4.36	4.42	4.49	4.46	4.55	4.58	4.73	4.69	4.04	4.41	4.53	4.51	4.71
Si/Al	1.86	1.91	1.83	1.91	1.87	1.86	1.85	1.88	1.86	1.93	1.94	1.88	1.86
K/Na	0.01	0.00	0.00	0.00	0.00	0.00	0.00	0.00	0.01	0.00	0.00	0.00	0.00

S7. (continue)

Sample	RS-8	RS-8	RS-8	RS-8	RS-8	RS-9	RS-9	RS-9	RS-9	RS-9	RS-9	RS-9	RS-9	RS-9	RS-9	RS-14	RS-14	RS-17	RS-17	RS-17
SiO_2	54.40	54.07	54.92	53.27	53.78	53.05	54.62	53.94	53.69	50.35	51.60	50.97	50.45	50.36	51.51	49.79	51.58	54.57	52.02	50.99
Al_2O_3	23.18	22.36	22.73	24.17	22.45	24.14	23.27	22.64	22.82	24.23	24.27	24.60	24.07	23.31	24.88	23.62	23.55	22.83	24.21	23.81
FeO**	0.08	0.08	0.12	0.07	-	0.29	0.17	0.02	0.35	0.16	0.28	0.29	0.36	0.29	0.09	0.39	0.32	0.13	0.26	0.19
MgO	0.16	0.00	0.04	0.11	-	0.12	0.07	-	0.09	0.09	0.05	0.12	0.16	0.12	0.08	0.50	0.07	-	-	-
CaO	0.84	1.34	0.50	2.35	0.52	1.93	0.55	0.53	1.09	3.07	2.35	3.04	2.81	2.32	2.47	1.89	0.93	0.90	3.34	2.91
Na_2O	12.84	12.83	13.12	12.39	12.87	12.48	13.16	12.90	12.61	10.56	12.14	12.10	10.64	11.58	12.02	12.07	12.93	13.39	11.09	11.63
K_2O	0.10	0.05	0.06	0.06	-	0.02	0.09	0.03	0.07	0.02	0.10	0.15	0.07	0.13	0.01	0.12	0.01	0.04	0.06	0.11
BaO	-	-	0.19	-	0.03	0.06	0.20	-	0.04	0.02	0.15	-	-	-	-	0.07	-	-	0.02	0.09
Total	91.60	90.73	91.68	92.42	89.65	92.09	91.93	90.06	90.76	88.50	90.94	91.27	88.56	88.11	91.06	88.45	89.39	91.86	91.00	89.73
H_2O*	8.40	9.27	8.32	7.58	10.35	7.91	8.07	9.94	9.24	11.50	9.06	8.73	11.44	11.89	8.94	11.55	10.61	8.14	9.00	10.27
										cations based on 96 oxygens										
Si	31.94	32.11	32.24	31.17	32.21	31.17	31.95	32.16	31.90	30.72	30.80	30.41	30.78	30.96	30.62	30.59	31.20	32.03	30.94	30.84
Al	16.04	15.65	15.72	16.67	15.85	16.71	16.04	15.91	15.98	17.42	17.07	17.29	17.31	16.89	17.43	17.10	16.79	15.79	16.97	16.97
Fe	0.04	0.04	0.06	0.03	0.00	0.14	0.08	0.01	0.17	0.08	0.14	0.14	0.18	0.15	0.04	0.20	0.16	0.06	0.13	0.10
Mg	0.14	0.00	0.03	0.10	0.00	0.11	0.06	0.00	0.08	0.08	0.04	0.11	0.15	0.11	0.07	0.46	0.06	0.00	0.00	0.00
Ca	0.53	0.85	0.31	1.47	0.33	1.21	0.34	0.34	0.69	2.01	1.50	1.94	1.84	1.53	1.57	1.24	0.60	0.57	2.13	1.89
Na	14.62	14.77	14.93	14.06	14.94	14.22	14.93	14.91	14.52	12.49	14.05	13.99	12.58	13.80	13.85	14.38	15.16	15.23	12.79	13.64
K	0.07	0.04	0.04	0.04	0.00	0.01	0.07	0.02	0.05	0.02	0.08	0.11	0.05	0.10	0.01	0.09	0.01	0.03	0.05	0.08
Ba	0.00	0.00	0.04	0.00	0.01	0.01	0.05	0.00	0.01	0.00	0.04	0.00	0.00	0.00	0.00	0.02	0.00	0.00	0.00	0.02
H_2O	16.44	18.36	16.28	14.79	20.67	15.49	15.74	19.76	18.30	23.39	18.03	17.36	23.27	24.37	17.72	23.66	21.40	15.93	17.84	20.71
Total	63.38	63.47	63.39	63.54	63.34	63.59	63.52	63.35	63.40	62.82	63.72	64.00	62.89	63.54	63.60	64.09	63.99	63.71	63.00	63.54
E%	0.08	-5.24	-0.24	-3.31	1.41	-1.08	0.93	1.90	-1.02	4.37	-1.25	-5.01	4.23	-1.69	1.65	-4.50	1.73	-3.70	-0.76	-3.22
CEC_{teor}	4.54	4.63	4.47	4.90	4.34	4.79	4.52	4.36	4.52	4.55	4.82	5.08	4.53	4.65	4.80	4.85	4.54	4.65	4.79	4.83
Si/Al	1.99	2.05	2.05	1.87	2.03	1.86	1.99	2.02	2.00	1.76	1.80	1.76	1.78	1.83	1.76	1.79	1.86	2.03	1.82	1.82
K/Na	0.00	0.00	0.00	0.00	0.01	0.00	0.00	0.00	0.00	0.00	0.00	0.00	0.00	0.00	0.01	0.01	0.00	0.01	0.00	0.01

*calculated by difference; ** otal Fe expressed as FeO; CECteor = theoretical Cation Exchange Capacity (expressed as meq/g)
