# Supplementary material for: Authigenic mineralization in Surtsey basaltic tuff deposits at 50 years after eruption
Source: Sci Rep. 2023 Dec 21;13:22855. doi: 10.1038/s41598-023-47439-4 (PMC10739796; doi:10.1038/s41598-023-47439-4)
Supplement: Supplementary file 8 — Supplementary Table S5. [file 41598_2023_47439_MOESM8_ESM.pdf]

S8. Chemical analyses for the investigated Al-tobermorite.

Sample	RS-2	RS-2	RS-2	RS-2	RS-2	RS-2	RS-2	RS-2	RS-2	RS-3	RS-3	RS-3	RS-3	RS-3
SiO_2	45.22	43.86	44.96	44.50	45.99	45.73	45.61	45.84	46.41	43.25	43.28	43.76	43.48	46.88
Al_2O_3	5.61	5.58	5.52	5.80	5.04	4.99	5.38	5.49	5.92	6.88	6.49	6.41	6.62	2.58
FeO**	0.16	0.18	0.22	0.20	0.28	0.27	0.25	0.27	0.29	0.04	0.06	0.31	-	0.12
MgO	0.03	-	0.02	-	-	0.07	0.06	0.05	0.02	-	0.03	0.02	0.06	-
CaO	34.93	33.19	34.75	35.20	35.67	32.91	33.97	34.79	36.45	32.16	34.64	34.86	33.83	36.71
Na_2O	0.09	0.02	0.05	0.16	0.13	0.22	0.15	0.06	0.07	0.24	0.22	0.05	0.11	0.12
K_2O	0.11	0.22	0.33	0.38	0.29	0.27	0.23	0.29	0.27	0.48	0.32	0.32	0.33	0.24
BaO	0.02	0.18	0.12	0.12	0.13	0.03	0.22	-	0.06	0.10	-	-	-	0.14
Total	86.17	83.23	85.97	86.36	87.53	84.49	85.87	86.79	89.49	83.15	85.04	85.73	84.43	86.79
H_2O*	13.83	16.77	14.03	13.64	12.47	15.51	14.13	13.21	10.51	16.85	14.96	14.27	15.57	13.21
								cations based on 16 oxygens						
Si	5.24	5.26	5.23	5.18	5.27	5.37	5.30	5.27	5.20	5.17	5.10	5.12	5.14	5.43
Al	0.77	0.79	0.76	0.79	0.68	0.69	0.74	0.74	0.78	0.97	0.90	0.88	0.92	0.35
Fe	0.02	0.02	0.02	0.02	0.03	0.03	0.02	0.03	0.03	0.00	0.01	0.03	0.00	0.01
Mg	0.01	0.00	0.00	0.00	0.00	0.01	0.01	0.01	0.00	0.00	0.01	0.00	0.01	0.00
Ca	4.33	4.26	4.33	4.39	4.38	4.14	4.23	4.28	4.37	4.12	4.38	4.37	4.29	4.56
Na	0.02	0.00	0.01	0.04	0.03	0.05	0.03	0.01	0.02	0.06	0.05	0.01	0.03	0.03
K	0.02	0.03	0.05	0.06	0.04	0.04	0.03	0.04	0.04	0.07	0.05	0.05	0.05	0.04
Ba	0.00	0.01	0.01	0.01	0.01	0.00	0.01	0.00	0.00	0.00	0.00	0.00	0.00	0.01
H_2O	5.34	6.70	5.45	5.29	4.76	6.07	5.47	5.06	3.92	6.72	5.88	5.57	6.14	5.10
Total	10.40	10.37	10.42	10.47	10.43	10.33	10.37	10.39	10.44	10.40	10.49	10.47	10.43	10.42

S8. (continue)

Sample	RS-3	RS-4	RS-4	RS-4	RS-4	RS-4	RS-4	RS-4	RS-4	RS-4	RS-8	RS-8
SiO_2	44.20	45.66	48.50	44.50	46.47	48.84	44.62	47.03	46.11	45.03	33.85	44.40
Al_2O_3	6.10	6.32	1.74	2.63	4.44	5.92	6.13	3.43	5.17	5.50	5.04	5.12
FeO**	0.35	0.32	0.19	0.06	0.25	0.16	0.09	0.04	0.26	0.19	0.17	0.12
MgO	0.04	0.10	-	0.10	0.04	0.05	0.09	0.02	0.05	0.13	0.07	-
CaO	34.64	36.00	37.52	39.51	36.33	37.41	36.07	37.00	35.78	35.35	24.52	35.18
Na_2O	0.36	0.24	0.12	0.13	0.09	0.16	0.06	0.09	0.38	0.50	0.29	0.24
K_2O	0.34	0.55	0.31	0.15	0.28	0.23	0.36	0.18	0.46	0.49	0.40	0.44
BaO	0.06	-	-	-	-	-	0.21	-	-	0.08	-	0.13
Total	86.09	89.19	88.38	87.08	87.90	92.77	87.63	87.79	88.21	87.27	64.34	85.63
H_2O*	13.91	10.81	11.62	12.92	12.10	7.23	12.37	12.21	11.79	12.73	35.66	14.37
						cations based on 16 oxygens						
Si	5.15	5.14	5.51	5.22	5.30	5.25	5.13	5.37	5.25	5.19	5.22	5.22
Al	0.84	0.84	0.23	0.36	0.60	0.75	0.83	0.46	0.69	0.75	0.92	0.71
Fe	0.03	0.03	0.02	0.01	0.02	0.01	0.01	0.00	0.02	0.02	0.02	0.01
Mg	0.01	0.02	0.00	0.02	0.01	0.01	0.02	0.00	0.01	0.02	0.02	0.00
Ca	4.33	4.34	4.57	4.97	4.44	4.31	4.44	4.53	4.36	4.36	4.05	4.43
Na	0.08	0.05	0.03	0.03	0.02	0.03	0.01	0.02	0.08	0.11	0.09	0.05
K	0.05	0.08	0.04	0.02	0.04	0.03	0.05	0.03	0.07	0.07	0.08	0.07
Ba	0.00	0.00	0.00	0.00	0.00	0.00	0.01	0.00	0.00	0.00	0.00	0.01
H_2O	5.41	4.06	4.40	5.05	4.60	2.59	4.74	4.65	4.47	4.89	18.35	5.63
Total	10.49	10.50	10.41	10.62	10.43	10.40	10.49	10.42	10.48	10.53	10.40	10.49

S8. (continue)

Sample	RS-8	RS-8	RS-8	RS-8	RS-8	RS-9	RS-9	RS-9	RS-14	RS-14
SiO_2	46.20	46.22	45.22	45.14	46.37	36.17	45.16	30.41	43.24	44.84
Al_2O_3	4.81	5.13	6.70	6.97	4.70	5.17	6.87	4.27	6.21	6.62
FeO**	-	0.19	0.16	-	0.21	0.07	0.46	0.14	0.19	-
MgO	0.06	0.02	-	0.03	0.02	0.01	0.09	0.14	0.01	0.04
CaO	35.80	36.07	34.88	35.04	36.46	26.85	34.24	21.87	33.98	35.72
Na_2O	0.05	0.16	0.47	0.31	0.23	0.70	1.00	0.48	0.79	0.80
K_2O	0.24	0.24	0.52	0.45	0.37	0.67	0.77	0.50	0.59	0.55
BaO	0.05	0.19	0.00	0.08	0.19	-	0.05	0.05	-	0.24
Total	87.21	88.22	87.95	88.02	88.55	69.64	88.64	57.86	85.01	88.81
H_2O*	12.79	11.78	12.05	11.98	11.45	30.36	11.36	42.14	14.99	11.19
					cations based on 16 oxygens					
Si	5.30	5.26	5.15	5.13	5.27	5.19	5.12	5.24	5.12	5.09
Al	0.65	0.69	0.90	0.93	0.63	0.87	0.92	0.87	0.87	0.89
Fe	0.00	0.02	0.02	0.00	0.02	0.01	0.04	0.02	0.02	0.00
Mg	0.01	0.00	0.00	0.01	0.00	0.00	0.02	0.04	0.00	0.01
Ca	4.40	4.39	4.25	4.27	4.44	4.13	4.16	4.03	4.31	4.34
Na	0.01	0.04	0.10	0.07	0.05	0.19	0.22	0.16	0.18	0.18
K	0.04	0.03	0.08	0.07	0.05	0.12	0.11	0.11	0.09	0.08
Ba	0.00	0.01	0.00	0.00	0.01	0.00	0.00	0.00	0.00	0.01
H_2O	4.89	4.47	4.57	4.54	4.34	14.54	4.29	24.19	5.92	4.24
Total	10.40	10.44	10.49	10.47	10.47	10.53	10.59	10.47	10.58	10.59

*calculated by difference; ** total Fe expressed as FeO
