# Supplementary material for: Authigenic mineralization in Surtsey basaltic tuff deposits at 50 years after eruption
Source: Sci Rep. 2023 Dec 21;13:22855. doi: 10.1038/s41598-023-47439-4 (PMC10739796; doi:10.1038/s41598-023-47439-4)
Supplement: Supplementary file 9 — Supplementary Table S6. [file 41598_2023_47439_MOESM9_ESM.pdf]

S9. Chemical analyses for the investigated gypsum.

Sample	RS-2	RS-4	RS-4	RS-3	RS-3	RS-3
SiO_2	0.42	0.11	0.15	0.09	0.05	0.08
TiO_2	-	-	0.08	0.06	-	0.11
Al_2O_3	0.14	0.09	0.09	0.06	-	0.03
FeO**	0.07	0.17	0.10	0.06	-	0.20
MnO	0.02	-	-	-	-	0.09
MgO	0.02	0.03	0.04	-	0.01	0.05
CaO	34.96	36.23	31.08	38.71	38.94	38.21
Na_2O	0.05	0.06	0.06	-	0.03	0.04
K_2O	0.06	0.04	0.02	-	0.03	0.11
BaO	-	0.02	-	-		
SO_3	50.77	52.26	45.28	57.10	57.90	56.42
P_2O_5	0.03	0.09	-	0.15	0.20	0.10
Total	86.54	89.10	76.90	96.23	97.16	95.44
H_2O*	13.46	10.90	23.10	3.77	2.84	4.56
			cation based on 4 oxygens			
Si	0.01	0.00	0.00	0.00	0.00	0.00
Ti	0.00	0.00	0.00	0.00	0.00	0.00
Al	0.00	0.00	0.00	0.00	0.00	0.00
Fe	0.00	0.00	0.00	0.00	0.00	0.00
Mn	0.00	0.00	0.00	0.00	0.00	0.00
Mg	0.00	0.00	0.00	0.00	0.00	0.00
Ca	0.98	0.99	0.98	0.97	0.97	0.97
Na	0.00	0.00	0.00	0.00	0.00	0.00
K	0.00	0.00	0.00	0.00	0.00	0.00
Ba	0.00	0.00	0.00	0.00	0.00	0.00
S	1.00	1.00	1.00	1.01	1.01	1.00
H_2O	1.17	0.93	2.26	0.29	0.22	0.36

*calculated by difference; **total Fe expressed as FeO
