# Supplementary material for: Authigenic mineralization in Surtsey basaltic tuff deposits at 50 years after eruption
Source: Sci Rep. 2023 Dec 21;13:22855. doi: 10.1038/s41598-023-47439-4 (PMC10739796; doi:10.1038/s41598-023-47439-4)
Supplement: Supplementary file 10 — Supplementary Table S7. [file 41598_2023_47439_MOESM10_ESM.pdf]

S10. Chemical analyses for the investigated calcite.

	RS-2	RS-3	RS-3	RS-3	RS-9	RS-9
SiO_2	0.20	2.69	5.48	6.48	0.19	1.90
Al_2O_3	0.12	0.46	0.89	1.16	0.06	0.27
FeO**	0.04	0.13	0.13	0.12	0.13	0.00
CaO	53.04	48.01	49.03	44.17	51.71	52.58
BaO	-	0.06	-	-	-	0.20
P_2O_5	-	0.02	-	-	-	-
SO_3	2.61	3.26	3.09	3.01	4.74	4.14
Total	56.01	54.63	58.62	54.94	56.83	59.09

** total Fe expressed as FeO
